# Education of household head and maternal healthcare utilization: the case of Bangladesh

**DOI:** 10.1186/s12889-024-20819-9

**Published:** 2024-12-18

**Authors:** Md Mahabubur Rahman, Md Tazvir Amin, Zannatul Ferdous, Hridoy Patwary, M. Moinuddin Haider

**Affiliations:** 1 Health Systems and Population Studies Division, icddr,b, GPO Box 128, Dhaka, 1000 Bangladesh; 2 Institute of Health Economics, Administrative Building (Ground Floor), University of Dhaka, Dhaka, 1000 Bangladesh; 3 Maternal and Child Health Division, icddr,b., GPO Box 128, Dhaka, 1000 Bangladesh

**Keywords:** Antenatal care, Facility birth, Household head, Adolescent, Bangladesh

## Abstract

**Background:**

The high maternal mortality ratio in South Asian countries could be attributed to poor maternal healthcare (MHC) utilization. Here household heads (HHs) are the main decision-makers of the households and thus can be key stakeholders in women’s MHC uptake. We aim to investigate the role of HHs’ education in MHC utilization and explore the educational status of male adolescents who will eventually replace today’s HHs in the future.

**Method:**

We investigated antenatal care (ANC), and institutional delivery as two MHC during pregnancy and childbirth using the Bangladesh Multiple Indicator Cluster Survey 2019. Due to the stratified cluster sampling nature of the BMICS 2019, we estimated odds ratios from mixed-effect multiple logistic regression considering women nested within clusters and estimated marginal effects to conclude. Using the Bangladesh Adolescent Health and Wellbeing Survey 2019–20, we estimated region-specific school dropout rates and identified the reasons for and timing of dropout among male adolescents.

**Results:**

The odds of ANC uptake and institutional delivery decreased by lower levels of HHs’ education. Marginal effects of HHs’ education on institutional delivery across comparable levels of ANC uptake show that less educated HHs diminish the full potential of ANC uptake in facilitating institutional delivery. The heaviest burden (~ 70%) of less educated (up to primary level) HHs was in the northeastern region. The highest rate of school dropout (40%) was also in the northeastern region. Around 60% of dropouts left school before or just after completing primary level. The primary reasons for dropout were lack of interest and financial constraints.

**Conclusion:**

Integrating HHs into MHC programs can be the immediate call for action while averting male adolescents’ school dropouts can be the long-term strategy.

**Supplementary Information:**

The online version contains supplementary material available at 10.1186/s12889-024-20819-9.

## Introduction

Southern Asian countries are still struggling to reduce their high maternal and neonatal mortality. They alone accounted for 20% and 38% of global maternal and neonatal deaths, respectively [1, 2]. Failure to prevent these deaths is a concern for human rights, public health, economy, and the development of these nations, which in fact jeopardizes the collective future of this globe [3, 4]. The leading causes of maternal death (postpartum hemorrhage, maternal infection, and eclampsia) and newborn death (birth asphyxia and pneumonia) suggest that adequate antenatal care (ANC) and institutional delivery services (IDS) could substantially prevent these deaths [5–10]. However, the ANC and IDS utilization remained far behind country-specific targets. There are supply-side barriers but many demand-side issues are yet to be resolved.

### Why household head?

Women’s participation in maternal health care (MHC) decision-making is low in patriarchal societies [11–14]. MCH decisions are primarily made by other influential persons in the household [11–14]. Being the main earner or the oldest male member of the household, the household head (HH) becomes the primary decision-maker of MHC in many cases [15]. Any healthcare decision-making process is a complex function of the decision maker’s knowledge about the health condition, ability to purchase the service, and empathy for the care-seeker. With the proposition that better education helps improve healthcare seeking, the education of the HH thus can have a promising role in women’s MHC utilization, especially in a patriarchal society.

### Household head- a bridge from ANC to facility births

Women of South Asian countries typically receive information on safe birth from ANC sessions. Usually, women are not accompanied by their HHs during ANC. So, the counseling on safe birth may not fully reach the most influential person in the household. The safe-birth-promoting message received by pregnant women may lose its potential if the HHs are not sensitized enough to recognize the relevance of the message. Hence, the less educated HHs could be a barrier to the benefits women could have gained through facility-birth-counseling during ANC.

### Male adolescents- the future household heads

While addressing the education of current HHs is crucial, it is equally important to consider the future HHs—today’s male adolescents. Ensuring the education of male adolescents is essential because it helps shape egalitarian attitudes, promotes gender equality, and improves healthcare-seeking behavior. If male adolescents become educated, they will be more likely to develop a sense of respect towards women and understand women’s necessity to use MHC. Hence, when they will become the HH, they may create an enabling environment in the household in favor of MHC usage. However, ensuring males’ education remains a challenge in low and middle-income countries.

### Bangladesh context

Bangladesh has the third largest population in South Asia [16]. Households are mainly headed by men ages 35 to 64 years [17]. The educational level of HHs is low; around one-third of the HHs never attended school, and one-quarter attended primary schools only [17]. Women’s working opportunities, and autonomy in their own healthcare are limited here [18]. With low levels of education among HHs and limited autonomy among women, MHC usage in Bangladesh remains inadequate. Only 37% of women take four or more ANC, and nearly half (49%) of births occur under institutional settings [17]. The low use of maternity care and facility birth partly contribute to the nightmare of a high maternal mortality ratio (196 maternal deaths per 100 thousand births) which has also remained unchanged since 2010 [19, 20].

### Earlier studies from Bangladesh

Previous studies from Bangladesh have shown that women’s education, media exposure, age at marriage, age at childbirth, parity, and socioeconomic status are associated with ANC and IDS uptake [21–23]. However, studies on the role of HH’s education attainment in MHC utilization are inadequate in Bangladesh [24, 25]. After reviewing thirty-eight MHC improving interventions implemented from 2000 to 2023, we found only one intervention that included HHs under the “community care” package (Appendix Table 1) [26]. Although the whole package had a positive impact, they did not examine the impact of integrating HH in improving MHC usage.

### Study aims and importance

Concerning the possible role of HHs’ education in MHC, suggesting HH-integrated intervention packages require evidence before implementation. Therefore, knowing whether the low level of HHs’ education is impeding women’s MHC usage in Bangladesh is of immense importance. To ensure the maximum benefits of ANC usage in promoting facility birth, it is crucial to know whether less educated HHs are diminishing the achievements of ANC. As education is still not universal in Bangladesh, investigating the schooling status of male adolescents is fundamental to understanding how future HHs are being prepared. However, none of the earlier studies shed light on the above aspects which are imperative to increase MHC uptake in Bangladesh.

Therefore, firstly we aim to investigate whether less educated HHs are impeding women’s ANC and IDS uptake and whether they are diminishing the maximum benefits of ANC usage in promoting facility birth. Furthermore, we attempted to estimate the rate and timing of, and reasons for school dropout among male adolescents, who will be the HHs tomorrow.

## Materials and methods

### Data

For objective 1: For assessing the first objective, we used the third round of the Bangladesh Multiple Indicator Cluster Survey (BMICS) 2019 data- the most recent publicly available nationwide dataset. The key variables collected in BMICS 2019 included housing characteristics, women and child’s sociodemographic characteristics, reproductive and maternal health indicators (ANC, facility birth, postnatal care), child health, nutrition and development (malnutrition, disease episodes, early childhood development) and etc. BMICS 2019 adopted a two-stage stratified cluster sampling technique making the data representative at national, division, district, and rural and urban level [17]. The urban and rural areas within each district formed the 128 strata from which 3220 clusters were chosen with probability proportional to size. Then a systematic sample of 20 households was selected from each cluster. BMICS 2019 was designed to provide estimates for a large number of indicators on the situation of children and women. BMICS interviewed 64,378 women from 61,242 households with a response rate of 93.7%. The key indicator used for calculating the sample size was the proportion of women with at least four ANC visits among women aged 15–49 years with a live birth in 2 years preceding the survey. BMICS considered the proportion to be 35% and survey estimate was 37% from a sample size of 9,285 which yields an estimated power to be 98%. This also yields a sufficient sample size for estimating other MHC indicators like facility birth, and post-natal care. The detailed sampling procedures of BMICS 2019 can be found in the survey report [17].

For objective 2: We explored the schooling status of male adolescents using data from the Bangladesh Adolescent Health and Wellbeing Survey (BAHWS) 2019–20- the first national-level survey on adolescents aged 15 to 19 in Bangladesh [27]. We included all 5,523 male adolescents aged 15 to 19 years interviewed in BAHWS 2019–20.

### Participants

For objective 1: The BMICS 2019 collected ANC and facility birth information from 9,285 women who had their last birth two years preceding the survey. As we aimed examining the role of HHs’ education on women’s MHC usage, women living in households where there are HHs who may influence women’s MHC uptake will be the target population. Women who themselves are the HHs will be independent from the influence of HHs and thus will not be in our target population. So, we excluded 333 women who were the head of their households. This exclusion will not introduce any biases to our estimates, because these women did not meet the criterion of target population.

ANC, facility birth, and education of HH were missing respectively for 5,1, and 3 women. As the sample size was sufficient enough for estimating ANC and facility birth, we excluded these 9 women from the analyses. Exclusion did not introduce potential bias in the estimates. If all these 9 women had 4 ANC visits, and facility birth, the estimated prevalences would change only at the second decimal (for ANC: without exclusion 36.96% vs after exclusion 36.89% and for facility birth: 53.54% vs 53.50%).

Finally, the analytical sample includes 8,943 ever-married women aged 15–49 years who gave birth two years preceding the survey.

### Outcome measures

For objective 1: The two primary outcomes of interest were at least four ANC uptake and IDS usage for the most recent birth, which we dichotomized in the following way:

1. At least four ANC visits: yes (if a woman had at least four ANC visits during her last childbirth), no (if a woman had no or at most three ANC visits during her last childbirth).
2. IDS usage: (if a woman gave her last birth in institutional settings other than home), no (if a woman gave her last birth at her, relatives’ or others’ home).

For objective 2: To explore the educational status of male adolescents we looked at three indicators: school dropout, educational level completed before school dropout, and reasons for dropout.

### Covariates

Covariates of interest: A pregnant woman is not the decision maker of MHC usage solely. Usually, HH controls the power dynamic of the household. Thus, if HHs are unaware of the benefits of MHC usage, they may not allocate the financial or human resources of the households for MHC usage. Educated HHs may have greater health literacy [28] and more egalitarian attitudes toward women’s autonomy in decision-making [25]. Earlier studies from Uganda and Bangladesh showed that HHs’ education helps improve MHC usage [25, 29]. Hence, keeping in mind the study objectives, the main covariate of interest was the education level of HH which we categorized as follows: none or pre-primary, primary, secondary, and above secondary.

Other covariates: We used Penchansky and Thomas’s Theory of Healthcare Access (ToHA) modified by Saurman [30] for other covariate selection. The six domains of the ToHA are accessibility, availability, acceptability, affordability, adequacy, and awareness. We conceptualized four broad groups of factors- household characteristics, women’s individual-level factors, women’s birth history, and contextual factors that may influence the six domains of the ToHA. Here one group of factors may influence more than one dimension of the ToHA. For example: the contextual factor “type of residence” can influence accessibility, availability, and acceptability domains respectively through transportation system, number of facilities, and cultural norms of using the facilities. Factors under each of the four broad groups were selected based on the earlier literature from Bangladesh [21, 24, 25, 30], India [31–34], Pakistan [35–38], and Nepal [35, 39–41]. Household characteristics include sex, age, religion of the HH; relationship with the HH; and household wealth status. Women’s individual-level factors comprise educational status, media exposure, and age at index birth. Women’s birth history contains the birth order and sex of the index child, and history of child death. Lastly, contextual factors include the place of residence and administrative division. We presented a detailed description of all the covariates in Appendix Table A.2.

Confounders: HHs’ gender; religion, wealth, and residence of the household; and women’s education have contextual rationale to be confounders. In Bangladesh context, male HHs are more educated than female HHs, but women from male-headed households are less likely to use MHC probably because male heads recognize women’s needs less than female heads do. Women from non-muslim, wealthy, urban households are more likely to have educated HHs and higher chances of MHC usage than the rest. Women’s education can also determine HHs’ education because educated women are more likely to be married in educated households. The positive role of women’s education in MHC usage is also recognized.

### Statistical analysis

Univariate statistics were used to understand the sociodemographic context of the study participants. Sampling weights and survey design characteristics of the corresponding survey were incorporated to reduce the bias from the estimates and produce robust standard error of the estimates. All the analyses were done using Stata version 14.0 (Stata SE 14, Stata Corp, College Station, TX, USA) and R (V.4.2.2, RStudio2023.09.0).

#### Analysis plan under objective 1

##### Model specification

BMICS 2019 used a cluster sampling design. Due to cluster-level heterogeneity in customs, beliefs, and infrastructural settings, MHC usage within a cluster can be correlated and so different clusters may start from different baseline levels. Hence, we used a mixed-effect logistic regression model considering random intercept at the cluster level to examine the association of HHs’ educational status with at least four ANC uptake and facility birth. This can deal with the hierarchical nature of the clustered data by incorporating both fixed effects and random effects at the cluster level. These random effects capture the variation across clusters by explicitly modeling the clustering structure and fixed effects represent the average relationship between the predictors and the response variable across all clusters.

If there is enough sample size, multivariable regression is a mathematical model that estimates an adjusted coefficient that controls for multiple confounders and covariates simultaneously [42]. Hence, we used multivariable regression models to adjust for potential confounders. The first model included only the main covariate of interest (HHs’ education). In the 2nd model, we added the confounders, and the 3rd model included other covariates as well. We performed sensitivity analyses for unmeasured confounding using the methodology proposed by VanderWeele and Ding in 2017 [43, 44]. We estimated the E-value, defined as the minimum strength of association, on the odds-ratio scale that an unmeasured confounder would need to have with both the exposure and the outcome to explain away the exposure-outcome association [45].

##### Marginal effect estimation

Further, we examined whether less educated HHs impede achieving the maximum benefits of ANC in facilitating institutional birth. For this, we estimated the marginal effects of HHs’ education on facility birth. In general, the marginal effect of X on the outcome (Y) is the instantaneous rate of change of Y with respect to X. For binary outcome with categorical covariate, the average marginal effect is the average change in outcome probability when a covariate changes from the reference category. For example: To estimate the marginal effects of a binary variable X, firstly one has to fit the regression model and estimate the predicted probability of the outcome for all the observed observations at the reference category (X = 0). Then subtract these from the predicted probability of outcome for all observed observations at the other category of X (X = 1). The average of these differences is the marginal effect of X on the outcome probability. So, the marginal effect equals 10 means the probability of the outcome will increase by 10% points among respondents with X = 1 than those with X = 0.

In our examination, we first constructed another mixed-effect multiple logistic regression considering random intercept at the cluster level with an interaction of HH’s education and the number of ANC visits. We estimated multiple logit-based marginal probabilities of institutional birth for each education level of HHs across the three groups of comparable number of ANC visits (No ANC visits, 1–3 ANC visits, 4 + ANC visits). Then, we estimated the marginal effects of HH’s education on institutional birth for the comparable number of ANC visits. These marginal effects allow concluding whether low education of HH diminishes the full-length benefits of ANC uptake in elevating institutional birth.

##### Spatial mapping

Lastly, we estimated the proportion of HHs with no education or primary education for each district and drew the spatial map of these district-wise percentages which allows for identifying the regions that may require immediate involvement of HHs in improving MHC usage.

#### Analysis plan under objective 2

Using BAHWS 2019–20 we estimated the percentage of school dropouts among male adolescents for each administrative division. Further, we explored the completed years of schooling of these dropouts by division which helps to understand the timing of quitting school. Lastly, we explored the reasons for quitting school.

## Results

### Sample characteristics

Table 1 presents the sociodemographic characteristics of the analytical sample. Around one-third of women had four ANC visits during their last childbirth and almost every second woman gave birth at home. Around 30% of women’s HHs were uneducated, and 30% had primary education. Two-thirds of the women’s HHs were their husbands. Households are primarily headed by men and half of the HHs were above 40 years old. About two-thirds of women had at least secondary level of education and around half of the women had media exposure almost every day.

**Table 1:** Sociodemographic characteristics of the analytical sample

Domains	Factors	%	Number of women
**Outcome of interest**	**Total**	100.0	8943
	**Number of ANC visits**		
	None	17.3	1,627
	1–3	45.8	4,112
	At least four	36.9	3,204
	**Place of birth**		
	Home	46.5	4,305
	Institution	53.5	4,638
**Household characteristics**	**Education of HH**		
	Above secondary	12.1	1,022
	Secondary	26.8	2,408
	Primary	29.8	2,725
	No or pre-primary	31.3	2,788
	**Relationship with HH**		
	Wife	67.4	6,018
	Daughter	2.7	246
	Daughter in law	27.0	2,443
	Other	2.9	236
	**Sex of HH**		
	Male	96.3	8,641
	Female	3.7	302
	**Age of HH**		
	< 30 years	16.25	1,409
	30–39 years	36.47	3,284
	40–49 years	17.2	1,532
	50–59 years	11.77	1,068
	60 years or above	18.32	1,650
	**Religion of HH**		
	Islam	91.6	8,067
	Other	8.4	876
	**Wealth status**		
	Poor	40.9	4,040
	Middle	18.9	1,727
	Rich	40.2	3,176
**Women’s characteristics**	**Women’s educational status**		
	No or primary	32.5	2,902
	Secondary	49.8	4,496
	Above secondary	17.6	1,545
	**Media exposure**		
	None	34.8	3,378
	Less than once a week	4.7	443
	At least once a week	8.3	762
	Almost everyday	52.2	4,360
	**Age at index childbirth**		
	< 18 years	6.9	599
	18–22 years	33.7	3,015
	23–27 years	28.9	2,588
	28 + years	30.5	2,741
**Women’s birth history**	**Death history of children born**		
	Had no death	91.8	8,219
	Had at least one death	8.2	724
	**Birth order of index child**		
	First	35.4	3,159
	Second	34.0	3,051
	Third or higher	30.6	2,733
	**Sex of index child**		
	Male	52.1	4,623
	Female	47.9	4,320
**Contextual factors**	**Place of residence**		
	Rural	77.9	7,223
	Urban	22.1	1,720
	**Administrative division**		
	Dhaka	24.0	1,716
	Barishal	5.6	807
	Chattogram	20.6	1,778
	Khulna	10.3	1,246
	Mymensingh	7.9	550
	Rajshahi	11.9	941
	Rangpur	11.2	1,129
	Sylhet	8.4	776

### Results under objective 1

**Fig. 1:**
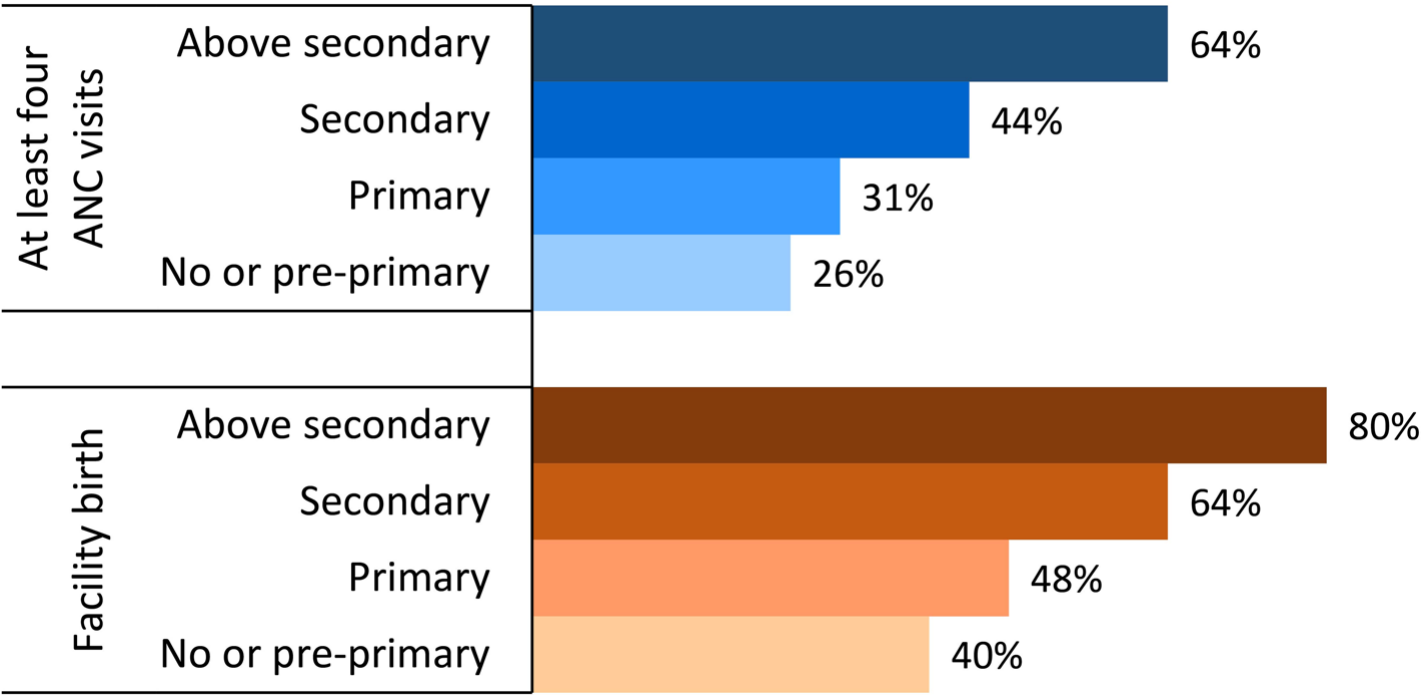
Prevalence of ANC uptake and facility birth by household heads’ education

***Is less educated HH a barrier to ANC uptake and IDS usage in Bangladesh?*** The prevalence of at least four ANC visits and institutional birth substantially declined as HHs’ educational attainment fell (Fig. 1). About 64% of women whose HHs had above secondary education attained at least four ANC sessions, while it was less than half respectively among women whose HHs had primary or no education. IDS usage was 80% when HH had above secondary level education, while it was only 40% among women whose HH had primary or no education. Appendix Table A.3 presents the prevalence of ANC uptake and IDS usage by other covariates.

Multivariable regression underscores a significant negative association of HHs’ education with women’s ANC uptake and institutional birth (Table 2). In comparison to women whose HHs had above secondary education, those with primary educated or uneducated heads had respectively 45% and 48% lower odds of completing four ANC visits. The odds of institutional birth were respectively 47% and 55% lower among women whose HHs had primary level or no education in reference to above secondary level educated HHs. Results of the full models are presented in Appendix Table A.4.

**Table 2:** Association of household heads’ education with women’s ANC uptake and institutional birth: Results from mixed-effect multivariable logistic regression

Factors	HHs’ education		HHs’ education + confounders	
	**UOR**	**95% CI**	**AOR**	**95% CI**
***At least four ANC visits***				
** Education of household head**				
Above secondary	Reference		Reference	
Secondary	0.41^***^	[0.30,0.56]	0.70^**^	[0.51,0.97]
Primary	0.22^***^	[0.16,0.31]	0.55^***^	[0.39,0.79]
No or pre-primary	0.19^***^	[0.13,0.26]	0.52^***^	[0.36,0.77]
Intra cluster correlation	24.3%		18.7%	
***Institutional birth***				
** Education of household head**				
Above secondary	Reference		Reference	
Secondary	0.38^***^	[0.26,0.56]	0.70^*^	[0.47,1.05]
Primary	0.19^***^	[0.13,0.27]	0.53^***^	[0.36,0.80]
No or pre-primary	0.14^***^	[0.10,0.20]	0.45^***^	[0.29,0.68]
Intra cluster correlation	29.0%		21.3%	

Note: *p-value < 0.10, **p-value < 0.05, ***p-value < 0.01, *UOR* Unadjusted odds ratio, *AOR* Adjusted odds ratio, *CI* Confidence interval, Result of the full model is presented in Appendix Table A.4 and Appendix Table A.5

For the confounder-adjusted ANC model, the estimated E-value for no or primary education was 2.12. So, an unmeasured confounder would need to have an odds ratio of 2.12 with HHs’ education and at least four ANC uptakes to nullify the association between HHs’ education and ANC. Similarly, for the institutional birth model, E-value was 2.35. So far authors’ understanding of the possible confounders for the association of HHs’ education with ANC and facility birth, there are no such confounders in addition to the adjusted ones (HHs’ gender; religion, wealth, and residence of the household; and women’s education) which might have a 2 times odds with HHs’ education and ANC and facility birth.

**Fig. 2:**
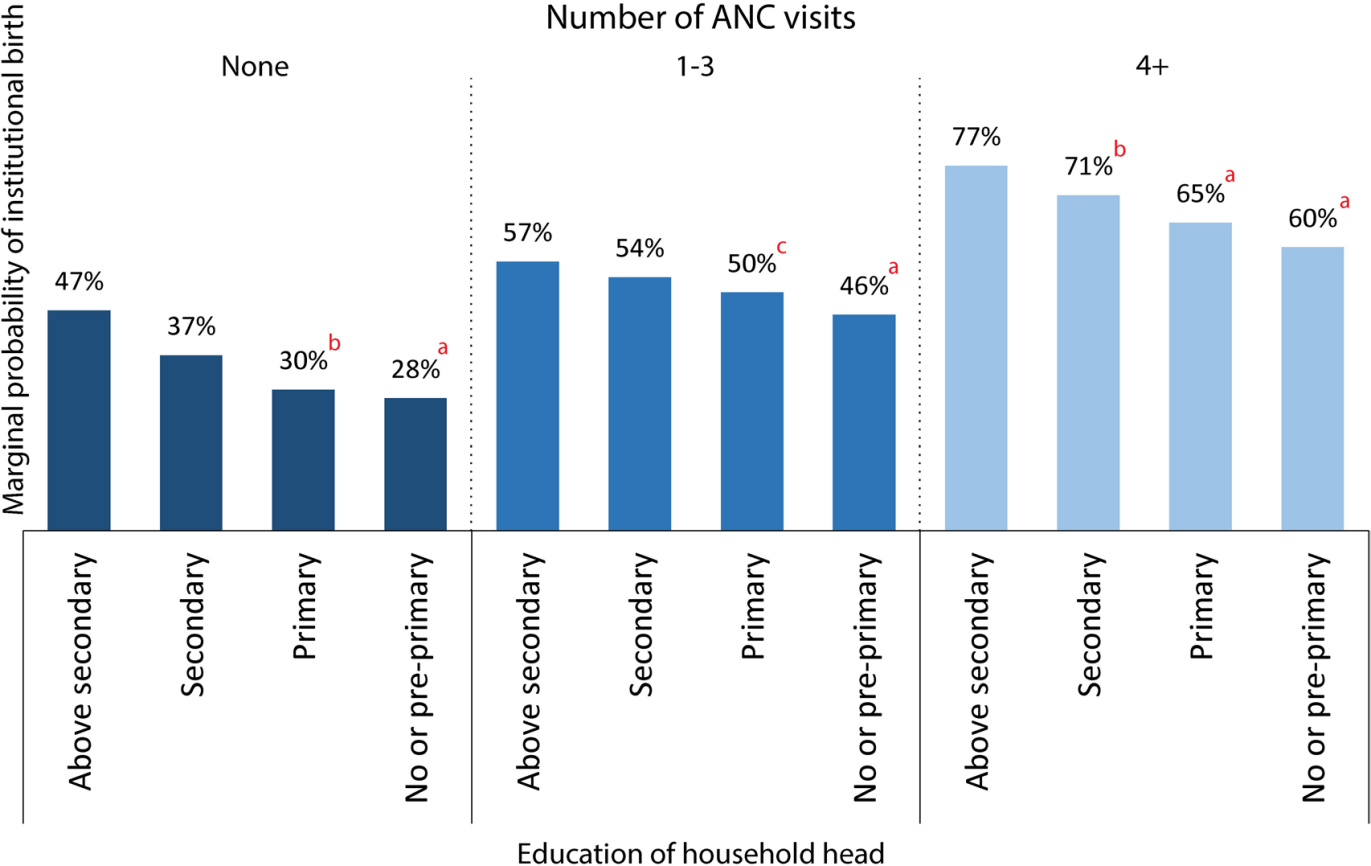
Probability of facility birth by number of ANC visits and education of household head from BMICS 2019. Note: c: *p* < 0.10, b: *p* < 0.05, a: *p* < 0.01

***Do less educated HHs diminish the full-length benefits of ANC on facility birth?*** Figure 2 presents the marginal probabilities of facility birth by HHs’ education for comparable levels of ANC visits. Figure 2 also shows the significance of the marginal effect of HHs’ education on facility birth across these ANC levels using superscript letters. The results of the regression model from which we estimated the marginal probabilities and marginal effects are presented in Appendix Table A.6. It seems that facility birth increases with the increased number of ANC visits. However, among women with comparable ANC visits, women from households with less educated heads were significantly less likely to have facility birth. The adverse effect of less education of HHs on facility birth remains even when there is an optimal number of ANC visits (above four visits). Of women who will have above four ANC visits but from households with no or pre-primary educated heads, 60% may have institutional birth (Fig. 2). However, it may reach 77% if the HHs have above secondary education. The maximum level of facility birth rate that we could reach by uplifting to a higher number of ANC visits significantly diminishes with each lower educational level of the HHs.

**Fig. 3:**
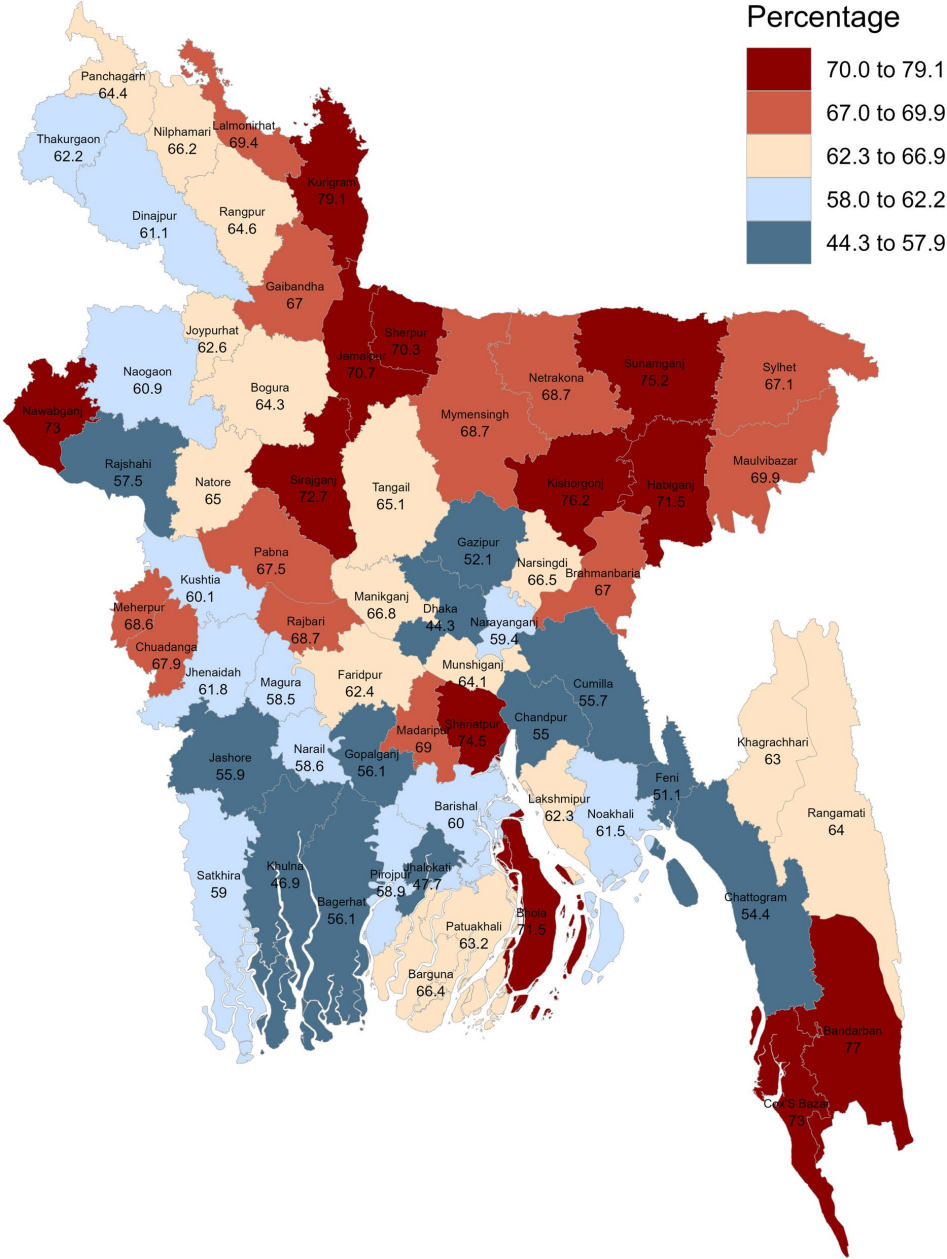
District-wise percentage of household heads with at most primary level education

***Which districts should undertake the HH engagement intervention?*** Figure 3 illustrates the district-wise percentage of HHs with low-level (no education or up to primary level) education. The educational level of HHs was the highest in Dhaka but roughly half of the heads had low-level education there which reflects the deplorable condition of education among HHs. The crisis is the most intensified in the northeastern region. In twelve districts, more than 70% of HHs had this low-level education, and in twelve more districts, it was close to 70%.

### Results under objective 2

**Fig. 4:**
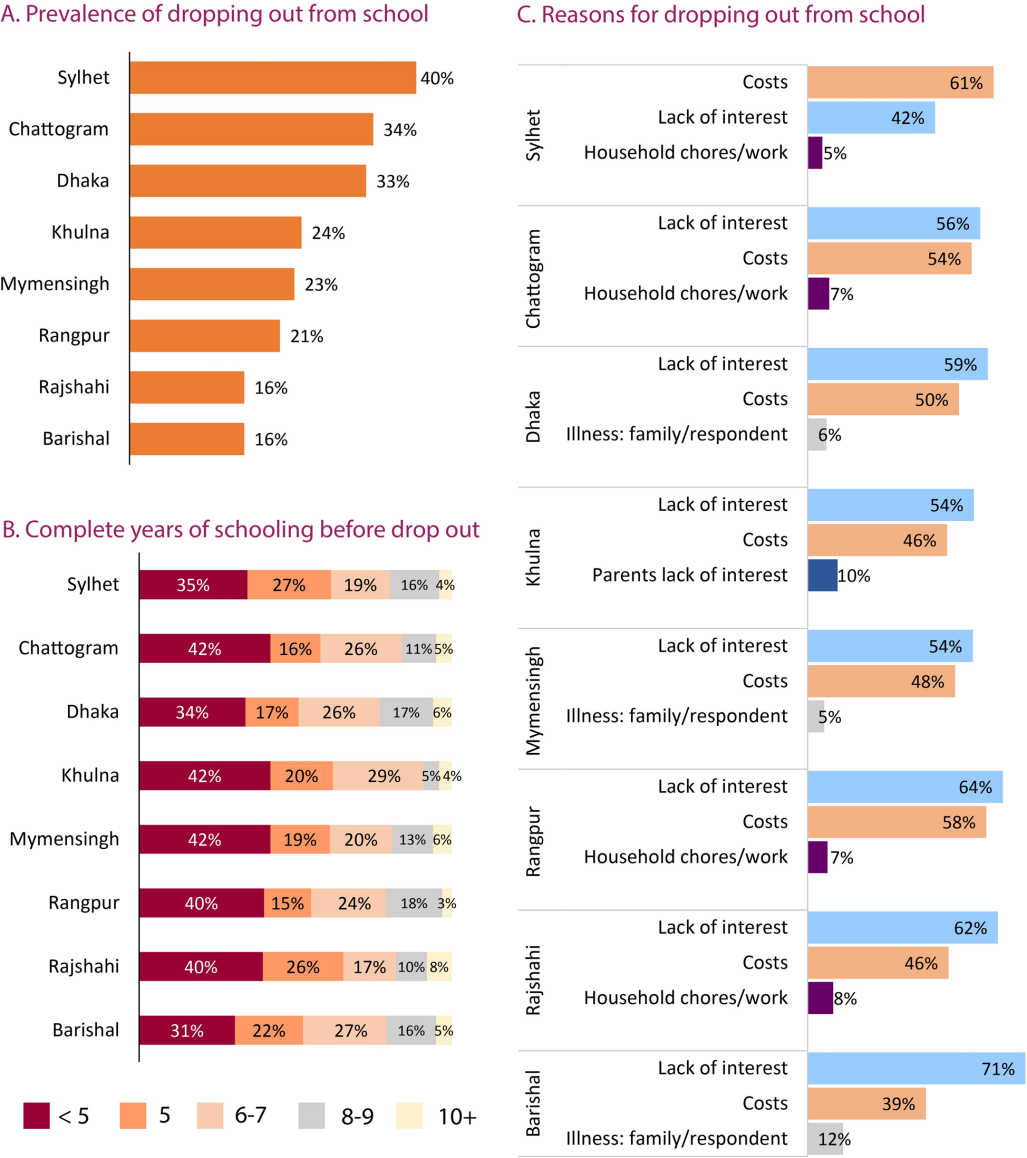
Prevalence, educational attainment and reasons for dropping out from school among male adolescents aged 15 to 19 years from BAHWS 2019–20

***In what proportion, when, and why do male adolescents drop out of school?*** A substantial variation in school dropout rate across divisions is visible in Fig. 4. The school dropout rate was the highest in the northeastern region (40% in Sylhet and 34% in Chattogram division). Timing of and reasons for dropping out of school remain similar across the divisions. More than half of the dropouts left school before or just after completing the primary level. Devastatingly, one-third of drop-outs left school even before completing the primary level. Lack of interest in education and the financial cost of schooling were the main reasons for dropping out. About 30% of the dropped outs who quit due to lack of interest also reported financial constraints. Household chores and the need to work for income were also found as barriers to schooling in some divisions.

## Discussion

### Main findings

This study finds that the higher the educational attainment of the HH, the better the MHC utilization. Even the low education of HHs diminishes the full-length benefits of ANC uptake in elevating the institutional birth rate. Low-educated HH is highly prevalent in the northeastern region of Bangladesh. In addition to the low education of current HHs, a future concern is the high school dropout of male adolescents (the next cohort of HHs), particularly in the northeastern region. Many left school before or just after primary completion. This indicates a high prevalence of HHs with low education in the future and warns about the low utilization of MHC among future mothers.

### ANC cannot play a full-length role in improving facility birth due to low education of household head

Four or more ANC visits enable facility birth. However, women living in households where the heads never attended school were 28% less likely to give birth in health facilities compared to women with HHs having above secondary education. Whereas low awareness about appropriate health care due to no education was one of the possible reasons, men’s inadequate involvement in maternal health programs is another reason that left the HHs with no or low education unsensitized. For example, the government’s MHC knowledge-transferring model (KTM) informs women about safe birth (e.g., facility birth) through the providers during ANC sessions [46]. As HHs generally do not accompany pregnant women at ANC, they remain outside the KTM program. The HH may receive the KTM messages from the pregnant women later, but that must not be as strong as it would be in his presence during the ANC session. We interpret the low involvement of HHs in MHC programs (intentional or unintentional) as a missing link between 4 + ANC and non-institutional birth.

### Challenges and opportunities to integrate household heads in MHC programs

In Bangladesh, face-to-face counseling is the most common form of intervention, followed by incentives and telehealth intervention packages. Engaging HHs through traditional counseling modalities such as household visits and courtyard meetings is less likely to function because HHs remain outside the home during working hours. Even after working hours, they spend considerable time in social gatherings in local markets, tea stalls, and other places instead of staying at home. Thus, rescheduled and relocated interventions can be an approach to ensure the engagement of HHs. Bangladesh has already introduced telehealth interventions for mothers to improve MHC usage [47–50]. However, these programs exclude HHs. The high coverage of at least one ANC opens the scope of collecting contact numbers of the HHs from pregnant women. Then, HHs can be informed and counseled on the benefits of MHC usage through interactive voice responses or voice messages, and the impact can be evaluated.

### Schooling of male adolescents to improve MHC

The high school dropout rates signal that the curse of the low education of HHs may not end soon, particularly in the northeastern region. The leading reasons for quitting school are financial constraints and lack of interest. A literature review of 44 school continuing interventions implemented between 2000 and 2023 indicates the inadequacy of incentives for boys, possibly explaining the financial constraint of discontinuing school (Appendix Figure A.1, Appendix Table A.7). BAHWS 2019 does not have the data that can explain the reasons for lack of interest. However, the lack of interest in continuing school rethinking the whole education system. From a simple eye lack of interest can be explained through uninteresting textbook content, monotonous teaching approach, and continuous unsatisfactory school performance. However, the main reasons for “lack of interest” may be rooted in the vicious cycle of low socioeconomic conditions. The intensified inequity in the quality between public and private educational institutions, and the cost associated with higher studies may diminish the hope of parents from disadvantageous groups. This may further shape parents’ efforts in building their children’s educational aspirations which may trigger the lack of interest in schooling among adolescents. Supporting this, we found that 30% of the droppedouts who quit due to lack of interest also reported financial constraints.

### Policy implications

#### Immediate call for action

Findings reveal that low-educated HHs are one of the barriers to women’s MHC usage, which remains unaddressed in almost all of the earlier interventions. As an immediate call for action, this study suggests an awareness program on MHC usage targeted at HHs. Mapping of HHs’ education signals the pressing need for such tailored awareness programs in the northeastern region. Besides northeastern districts, Kurigram, Bandarban, and Cox’s Bazar also demand especial attention. Evidence of the success of earlier interventions suggests telehealth as a viable strategy to reach the HHs.

#### Long-term policy actions

Findings suggest that MHC usage significantly increases if the HHs have at least secondary-level education. On the other hand, we found a high school dropout rate among male adolescents and around sixty percent of dropouts left school before enrolling in secondary grades. These suggest developing long-term policy actions to keep male adolescents at school until completing the secondary level of education. Findings suggest designing education programs targeted at the northeastern region. Because, in addition to the vulnerable situation of HHs’ education, this region is also suffering from the highest school dropout rates. We suggest the Government of Bangladesh to increase the coverage and thickness of the incentive programs and make the learning process interesting to keep the students motivated. Moreover, health, education, and finance ministries should collaborate on projecting the minimum percentage of the national budget that can be invested in HHs and male adolescents to meet the national maternal health strategy for achieving SDG.

### Limitation

Establishing any causal relationship is beyond the scope of this study. Any unmeasured confounders other than the adjusted ones which have a 2 times odds with HHs’ education and ANC and facility birth may explain away the association of HHs’ education with at least four ANC uptake and facility birth. From the data, we do not know who decided the place of birth. However, from BMMS 2016, we estimated that the HH decided every second facility birth. Answering why, when, and how the low-educated HH restricts women from facility birth was not possible using secondary data. This study recommends further investigation revealing the mechanisms by which low-educated HH lessen MHC usage. Our findings reveal “lack of interest” as one of the primary reasons for school dropout among male adolescents. However, data constraint restricts us from exploring the reasons for lack of interest, which is essential for designing interventions.

## Conclusion

Education of HHs significantly influences women’s MHC usage. The positive effect of 4 + ANC in facility delivery fades in households headed by low-educated persons. The findings signify the need to integrate HHs into MHC-improving programs. School dropout among male adolescents (future HHs) is high, due to financial constraints and lack of interest. In addition to the MHC programs, the education programs must identify strategies to keep boys in school.

## Supplementary Information

Additional file 1: Figure A.1 Interventions implemented during 2000 to 2023 to improve school enrollment. Table A.1 Interventions implemented during 2000 to 2023 to improve maternal healthcare services utilization. Table A.2 Description of the covariates. Table A.3 Prevalence of antenatal care (ANC) uptake and institutional birth across sociodemographic factors. Table A.4 Association of household head’s education with at least four ANC uptake: Results from mixed-effect multivariable logistic regression. Table A.5 Association of household head’s education with at facility birth: Results from mixed-effect multivariable logistic regression. Table A.6 Interaction of household head's education with number of ANC visits. Table A.7 Interventions implemented during 2000 to 2023 to improve school enrollment.

## Data Availability

BMICS 2019 is publicly available on the MICS website. The BAHWS 2019–20 data is publicly available at UNC dataverse.
