# Supplementary material for: Education of household head and maternal healthcare utilization: the case of Bangladesh
Source: BMC Public Health. 2024 Dec 18;24:3439. doi: 10.1186/s12889-024-20819-9 (PMC11654065; doi:10.1186/s12889-024-20819-9)
Supplement: Supplementary file 1 — Additional file 1: Figure A.1 Interventions implemented during 2000 to 2023 to improve school enrollment. Table A.1 Interventions implemented during 2000 to 2023 to improve maternal healthcare services utilization. Table A.2 Description of the covariates. Table A.3 Prevalence of antenatal care (ANC) uptake and institutional birth across sociodemographic factors. Table A.4 Association of household head’s education with at least four ANC uptake: Results from mixed-effect multivariable logistic regression. Table A.5 Association of household head’s education with at facility birth: Results from mixed-effect multivariable logistic regression. Table A.6 Interaction of household head's education with number of ANC visits. Table A.7 Interventions implemented during 2000 to 2023 to improve school enrollment. [file 12889_2024_20819_MOESM1_ESM.docx]

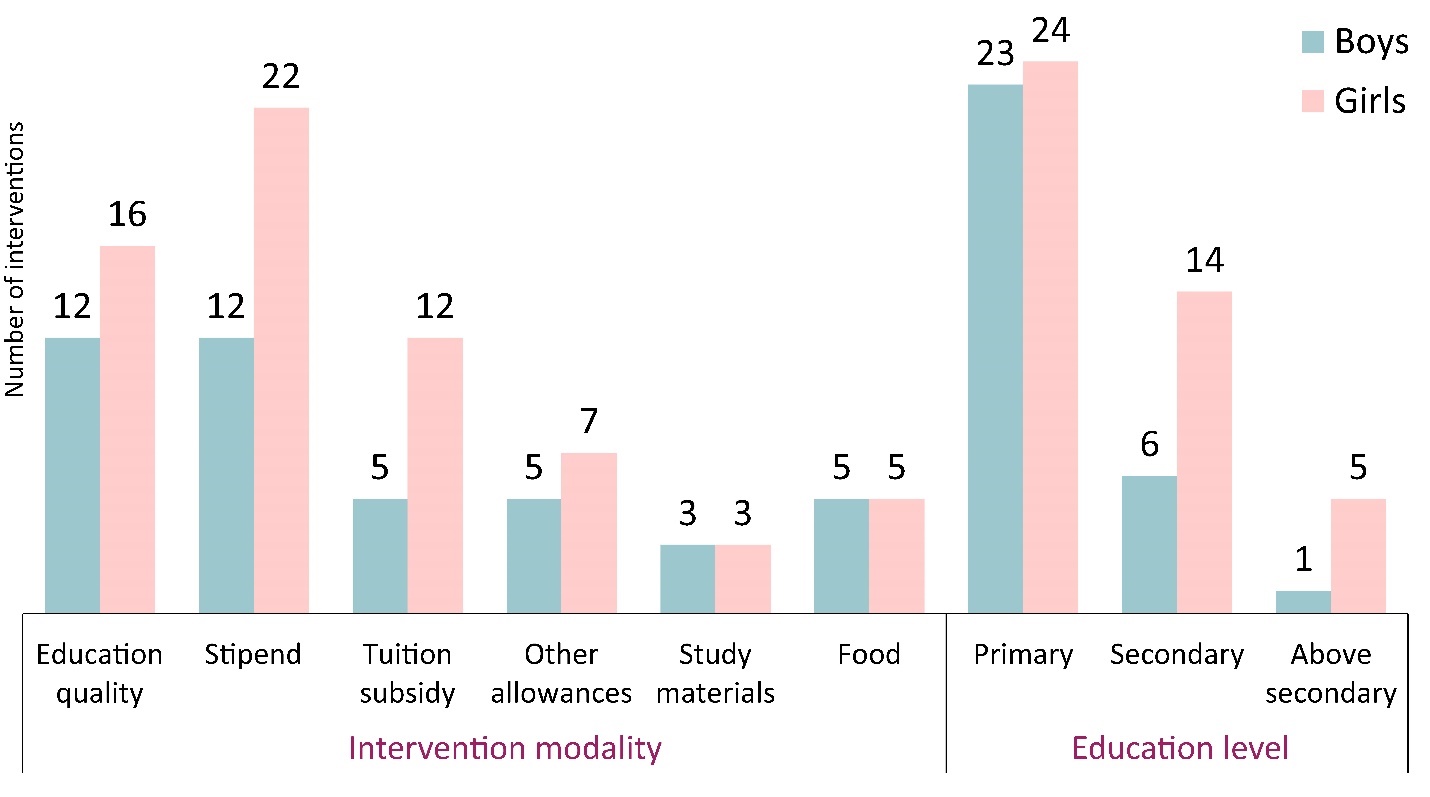


**Figure A.1 Interventions implemented during 2000 to 2023 to improve school enrollment**

**Table A.1 Interventions implemented during 2000 to 2023 to improve maternal healthcare services utilization**

| **Serial** | **Program Period** | **Title of the Program** | **Indicator** | **Type of Intervention** | **Intervention Recepient** | **Place** | **Source** |
| --- | --- | --- | --- | --- | --- | --- | --- |
| 1 | 2006-2011 | Safe Motherhood Promotion Project (SMPP) (Phase 1) | Maternal and neonatal health care | a. Training b. Meeting | a. Hospital Staffs (Nurses, FWVs, CSBA etc.) b. Women b2. husband c. Family members d. Community people | a. Hospital b. Courtyard | a. https://www.jica.go.jp/Resource/project/bangladesh/002/materials/ku57pq00001gtcss-att/narsingdi_model_in_bangladesh.pdf b. https://www.jica.go.jp/Resource/project/english/bangladesh/0602298/04/pdf/Report_2010_02.pdf c. https://journals.plos.org/plosone/article?id=10.1371/journal.pone.0212847 |
| 2 | 2007-2011 | MANOSHI program | ANC, PNC, skilled assisted delivery | a. Face-to-face counseling (FFC) b. Group counselling (GC) c. Mass media (MM) (Folk song performance; street dramas, TV spots and billboards) d. Training | a. Women b. Husband c. Family members b. CHWs | a. Household b. Health facilities | a. https://journals.plos.org/plosone/article?id=10.1371/journal.pone.0162825 b. https://brac.net/sites/default/files/portals/Manoshi-book-v3-1.pdf c. https://resource-allocation.biomedcentral.com/articles/10.1186/1478-7547-11-28 |
| 3 | 2008-2013 | Maternal and Neonatal Health Initiatives in Bangladesh (MNHIB) program | ANC, PNC, facility-based delivery | Counselling | a. Women b. Family members c. Community people | Courtyard | a. https://www.mhtf.org/2017/09/08/how-bangladesh-increased-maternal-health-care-utilization-and-reduced-inequities/ b. https://www.ncbi.nlm.nih.gov/pmc/articles/PMC5526556/ |
| 4 | 2006-2009 | Access to Clinical and Community Maternal, Neonatal and Women’s Health Services (ACCESS) Bangladesh Program | Safe delivery and a healthy outcome for mother and baby | Counselling | a. Mother-to-be b. Family members c. Other support persons d. Community people (Women's and men's groups) | a. Household b. Courtyard | a. https://www.jhpiego.org/accesstohealth/ b. https://pdf.usaid.gov/pdf_docs/PDACU778.pdf |
| 5 | 2009-2014 | MaMoni –Integrated Safe Motherhood, Newborn Care, and Family Planning Project | ANC, PNC, facility-based delivery, referrals for maternal complications, family planning (FP) services | a. Counseling b. Training | a. Women b. Family members c. Community people | a. Courtyard b. Health facilities | https://pdf.usaid.gov/pdf_docs/PA00K2MZ.pdf |
| 6 | 2009-2013 | Mayer Hashi project | ANC, PNC, infant immunization, family planning services | a. One-to-one meetings b. Group discussion c. Training d. Mass media (Flipchart, Interactive Guidebook, Roman Banner, and LA/PM Leaflet, Street Drama) | a. Young women & men b. Healthcare providers c. Religious and community leaders d. MOH&FW officials e. Community people | a. Household b. Courtyard b. Health facilities | a. https://pdf.usaid.gov/pdf_docs/PA00JN7F.pdf b. https://www.bangladesh-ccp.org/project/xergvkos/mayer-hashi-program |
| 7 | 2004-2010 | Strengthening Household Ability to Respond to Development Opportunities (SHOUHARDO) Program (Phase I) | ANC, PNC, birth attendant training, supplemental vitamins, family planning, vaccinations, diarrhea monitoring, and referral systems for emergencies | a. Training b. Group discussion c. Counselling | a. Pregnant women and women with children under 2 years (poor & extra poor) b. Adolescent girl b. Community people | a. Household b. Courtyard b. Health facilities d. Food ration | a. https://usaidlearninglab.org/sites/default/files/resource/files/cla_case_competition_casestory_23_care_shouhardo2_bangladesh_final.pdf b. https://www.care-international.org/sites/default/files/files/publications/MDG-Summit-CARE-Brief-Improve-Maternal-Health-2010.pdf |
| 8 | 2007-2015 | Maternal Health Voucher Scheme | Maternal and neonatal health care | Vouchers for free maternal health services (ANC, PNC), transport subsidies, and a cash incentive for giving birth with the assistance of a qualified maternal healthcare provider (public, non-governmental or private) and a gift box | Poor and vulnerable pregnant women | Monthly meeting | a. https://academic.oup.com/heapol/article/26/1/25/626276 b. https://reliefweb.int/report/bangladesh/demand-side-financing-bolsters-maternal-health |
| 9 | 2002-2006 | Community-Based Intervention to Reduce Neonatal Mortality in Bangladesh (Projahnmo - I) | Maternal nutrition, ANC, emergency obstetric care preparedness, skilled birth attendant and essential newborn care | a. Health education and counseling b. Provision of essential maternal and newborn care c. Strengthening of existing government health facilities and capacity building | a. Pregnant Women b. Family members c. husband d. household head | a. Household b. Community meetings | <https://chwcentral.org/wp-content/uploads/2013/07/Community-Based-Intervention-to-Reduce-Neonatal-Morality-in-Bangladesh.pdf> |
| 10 | 1998-2005 | Urban Primary Health Care Services Delivery Project (Phase-I) | ANC, PNC, child health care, health & nutrition education | a. Counseling b. Free treatment & medicine | Urban people (especially poor women & children) | a. Household b. Health facilities | a. https://www.adb.org/projects/42177-013/main b. http://uphcp.gov.bd/cmsfiles/files/Health%20Facility%20Survey%20Report(4).pdf |
| 11 | 2005-2012 | Urban Primary Health Care Services Delivery Project (Phase-II) | ANC, PNC, child health care, health & nutrition education | a. Counseling b. Free treatment & medicine c. Mass media interventions (miking, posters, leaflets, display of ad in the scroll message given by local cable channels, live shows, 13-episode TV drama series etc. ) | Urban people (especially poor women & children) | a. Household b. Health facilities c. Public places | a. https://www.adb.org/projects/42177-013/main b. https://www.bangladesh-ccp.org/project/lfrx2x69/urban-primary-health-care-services-project-uphcp-ii |
| 12 | 2009-2016 | A community-based intervention package in Netrokona | Maternal and newborn health | a. Promotion of birth preparedness and complication readiness (BPCR) b. Increasing awareness of rights and MNH needs through courtyard meetings c. Counselling by health care providers | a. Women b. Husband c. Family members d. Community people | a. Household b. Courtyard | https://www.ncbi.nlm.nih.gov/pmc/articles/PMC6318832/#R13 |
| 13 | 2008-2013 | Improved Maternal Newborn and Child Survival (IMNCS) project | Family planning, ANC, delivery care, PNC, essential neonatal care (ENC), neonatal illnesses, under-5 child illnesses, vaccination, referral for complications of mothers and children | a. Training b. Counselling | a. Women b. Community health workers | a. Household | https://journals.plos.org/plosone/article?id=10.1371/journal.pone.0136898#:~:text=The%20project%20for%20improving%20maternal,BRAC's%20frontline%20community%20health%20workers. |
| 14 | 2007-2009 | Maternal, Neonatal, and Child Health (MNCH) project in Matlab | Family planning, ANC, facility birth, PNC, essential neonatal care (ENC), breastfeeding | Counselling | a. Women b. Family members | Houshold | https://bmcpublichealth.biomedcentral.com/articles/10.1186/1471-2458-11-914 |
| 15 | 2008-2012 | Maternal, Neonatal and Child Survival Interventions (MNCS) | ANC, PNC, pregnancy complications, facility delivery | a. Training b. Counselling | a. Women b. Family members c. CHWs, public and private health care providers d. Local government bodies, village elites, school teachers and other community influencers | a. Household b. Health Facilities | https://www.researchgate.net/profile/Akm-Ahsan-Ullah/publication/357553763_Reduction_in_Lifetime_Fertility_Through_MNCS_in_Rural_Bangladesh/links/61d3d065e669ee0f5c83ae56/Reduction-in-Lifetime-Fertility-Through-MNCS-in-Rural-Bangladesh.pdf |
| 16 | 2009-2011 | A toll free mobile phone intervention | Skilled maternal health care service (maternal and neonatal complications) | A toll free mobile pathway among mothers, Community Based Skilled Providers (CSBAs) and specialized maternal health providers | a. Women b. Husband c. Health care providers |  | https://reproductive-health-journal.biomedcentral.com/articles/10.1186/1742-4755-11-52 |
| 17 | 2011-2016 | Safe Motherhood Promotion Project (SMPP) (Phase 2) | Maternal and neonatal health care | a. Training b. Counselling c. Capacity building d. ANC, PNC campaign  e. Local level advocacy f. Establish Community Support Group (CSG) f. Linkage between facilities and community | A. Women b. Family member c. CSG members, community volunteers d. Local government bodies, NGO members | a. Household b. Health Facilities | a. https://careevaluations.org/evaluation/terminal-evaluation-of-safe-motherhood-promotion-project-phase-ii/ b. https://www.jica.go.jp/Resource/project/english/bangladesh/002/materials/c8h0vm00004eggs3-att/briefing_document_01.pdf c. https://bmcpublichealth.biomedcentral.com/articles/10.1186/s12889-018-5478-6 |
| 18 | 2009-2014 | Healthy Child and Mother Project (SUSOMA) | Maternal and neonatal health care | a. Training b. Counselling c. Group disscussion d. Establish village level primary groups (Women/Men) e. Establish emergency health funds f. Transport systems, & active referral system | a. Women (15-49 years) b. Men c. Children under 5 years d. Health services providers e. Local NGO workers f. Community volunteers | a. Household b. Community c. Health Facilities | https://www.mcsprogram.org/wp-content/uploads/2015/08/World-Renew-Bangladesh-FE-Brief.pdf |
| 19 | 2019- | "Mother assembl" in Netrokona | ANC, PNC, Institutional delivery | a. Counselling c. Referral system d. Financial assistance | a. Pregnant mothers b. Children under 5 years | a. Courtyard b. Community certers | https://www.dhakatribune.com/bangladesh/181887/government-initiative-to-reduce-maternal-mortality |
| 20 | 2021- | Providing Comprehensive, Safe and Appropriate Intervention to Prevent Postpartum Hemorrhage for Reducing Maternal Mortality in Bangladesh | Postpartum Hemorrhage | a. Awareness building b. Technical and capacity building activities c. Distribution of misoprostol tablets and postnatal care kits | a. Pregnant mothers (remote rural communities) b. Health care providers |  | https://www.partners-popdev.org/press-release-virtual-launching-ceremony-of-the-south-south-cooperation-project-between-the-government-of-bangladesh-and-the-government-of-china-aimed-at-providing-comprehensive-safe-and-appropriate/ |
| 21 | 2017-2022 | Suchana | Maternal & child nutrition, ANC, PNC | a. Household counselling b. Courtyard meeting c. Growth monitoring and promotion (GMP) sessions d. Cooking and feeding demonstrations | a. Pregnant women b. Lactating mothers with children under 2 years c. Adolescent girls | a. Household b. Courtyard | a. https://www.eeas.europa.eu/delegations/bangladesh/suchana-ending-cycle-undernutrition-bangladesh_und_en b. https://onlinelibrary.wiley.com/doi/full/10.1111/mcn.13258 |
| 22 | 2015-2022 | Income Support Program for the Poorest (ISPP)-Jawtno (care in Bangla) | Children’s nutrition, cognitive development, and readiness for school | Conditional cash payments | a. Poor pregnant women b. Mothers with children under five years | Health Facilities | https://reliefweb.int/report/bangladesh/results-brief-supporting-mothers-and-helping-give-poor-children-bangladesh-better-start-life?gad_source=1&gclid=Cj0KCQiA3uGqBhDdARIsAFeJ5r1r2OWmQSQk5rZruICWdd7kx24uXUeFIdN_y2pevVlCHSv9Yyfx3tkaAnhYEALw_wcB |
| 23 | 2011-2019 | Lactating Mother Assistance Fund Program | Pregnancy health care, safe motherhood, health of mother and child, birth control, teaching the child, child marriage, dowry, women’s torture and mental development of children 0 to 5 years | a. Allowance b. Training c. Health camp with free medical services | Women of BKMEA's member factories | BKMEA's member factories | https://www.bkmea.com/projects/lactating-mother/#:~:text=Under%20this%20program%20each%20beneficiary,bank%20account%20as%20an%20allowance. |
| 24 | 2018-2026 | Mother and Children Benefit Programme (MCBP) | Mother & child nutrition, ANC, PNC, safe birth, children cognitive development | a. Allowance b. Social and behavior change communication (SBCC) session c. Counselling d. Food basket | a. Pregnant women and mothers of children under 4 years of age (vulnerable households) b. Family members | a. Mobile wallet or bank account of beneficiaries through electronic fund transfer under government to person payment system b. Health facilities c. Courtyard | a. https://www.unescap.org/sites/default/files/Item2_Bangladesh.pdf b. https://dwa.portal.gov.bd/sites/default/files/files/dwa.portal.gov.bd/miscellaneous_info/46117493_07a1_40d3_9456_e84b9b6c11e8/2020-04-28-15-14-94511cdd4e1225892ab8a687e19bb674.pdf c. https://www.unicef.org/bangladesh/en/media/8421/file/Scaling%20up%20for%20the%20Mother%20and%20Child%20Benefit%20Programme.pdf d. https://www.worldbank.org/en/news/press-release/2023/10/31/world-bank-helps-bangladesh-improve-early-childhood-development-benefitting-1-7-million-vulnerable-pregnant-women-and-mo e. https://bangladesh.ifpri.info/2023/05/new-blog-stepping-into-the-field-observing-bangladeshs-national-mother-and-child-benefit-program-to-inform-ifpri-research/ |
| 25 | 2020-cont. | Ma-Telehealth’ service | Maternal and child health care | a. Telehealth’ service (health care & counselling) b. Arranging necessary treatment, and coordinating with the health center | a. Pregnant women b. Lactating mothers b. Children aged 0-2 year | Through mobile | a. https://thefinancialexpress.com.bd/health/ma-telehealth-center-launched-1600268723 b. https://www.undp.org/bangladesh/stories/ma-telehealth%E2%80%99-service-emerges-blessing-pregnant-women |
| 26 | 2018-2023 | MaMoni Maternal and Newborn Care Strengthening Project (MNCSP) | Maternal and Newborn Health (MNH) Care | a. Training b. Counselling c. Provision of skilled providers, commodities, medical supplies and equipment d. mHealth based SMS and OBD reminder service e. Facilitated Referral Transport Model (FRTM) f. OpenMRS+ based registration, queue and reporting management system g. KIOSK based client feedback mechanism | a. Mothers, newborns and their family members b. Government health service providers, supervisors and managers, community level structures, local government institutions and private sector | Health Facilities | a. https://pdf.usaid.gov/pdf_docs/PA00ZHXQ.pdf b. https://dnet.org.bd/work/mamoni-maternal-and-newborn-care-strengthening-project-mamoni-mncsp/ c. https://resourcecentre.savethechildren.net/pdf/MaMoni-MNCSP-Project-Brief-updated-2021_English.pdf/ |
| 27 | 2015-2020 | Cash incentive program | ANC, child nutrition & knowlwdge development | Cash incentive | a. Poor pregnant women b. Family of children (0-5 years) | Health facilities | https://bdnews24.com/bangladesh/bangladesh-introduces-cash-incentive-for-poor-pregnant-women-and-children |
| 28 | 2018-2027 | mCARE program | Pregnancy surveillance and care-seeking reminders (ANC, child delivery and PNC) | a. Text messages b. Home visit c. Pregnancy surveillance using a mobile phone-based system | a. Pregnant women b. Family members | a. Household b. Through mobile | https://bmjopen.bmj.com/content/bmjopen/11/4/e042553.full.pdf |
| 29 | 2015-2022 | Strengthening Household Ability to Respond to Development Opportunities (SHOUHARDO) III Program | Maternal & child nutrition, institutional delivery, birth preparedness planning | a. Food rations (wheat, Vitamin A -fortified vegetable oil, yellow split peas)  b. Counselling c. Home visit d. Provide skilled assistance for uncomplicated births | a. Pregnant women (poor & extra poor) b. Lactationg mothers (poor & extra poor) c. Children under years | a. Courtyard b. Household | a. https://shouhardo.carebangladesh.org/en_US/ b. https://careclimatechange.org/wp-content/uploads/2019/03/SHOUHARDO-III-Booklet.pdf c. https://www.carebangladesh.org/media-center-view-details.php?type=Story&id=122 |
| 30 | 2018-2023 | JANO (Joint Action for Nutrition Outcome) | Maternal & child nutrition | a. Counselling b. Home visit c. Mass media intervention using pico projector | a. Pregnant and lactating women b. Adolescent girls | a. Courtyard b. Household | a. https://www.carebangladesh.org/publication/nutrition/JANONewsletter.pdf b. https://www.carebangladesh.org/publication/nutrition/JANO_BRIEF_FINAL.pdf |
| 31 | 2012-2017 | The Strengthening Partnerships, Results, and Innovations in Nutrition Globally (SPRING) project | Maternal & child nutrition | a. Counselling b. Home visit c. Capacity building | a. Pregnant and lactating women b. Children (0-2 years) c. Cummunity health workers | a. Courtyard b. Household c. Health facilities | https://www.spring-nutrition.org/sites/default/files/publications/reports/spring_bangladesh_final_country_report_508.pd |
| 32 | 2011-2016 | BBC Agomoni | ANC, birth preparedness, safe delivery, nutrition and essential newborn care | a. TV drama-Ujan Ganger Naiya (Sailing Against the Tide), a discussion show, four ringtones, public service announcements, outreach activities including screenings of the drama in communities b. Training | a. Women of reproductive age b. Mother-in-law c. Father-in-law d. Husband e. Health workers | a. Mass media b. Public place | https://www.bbc.co.uk/mediaaction/where-we-work/asia/bangladesh/mch |
| 33 | 2018-2023 | Sustained Opportunities for Nutrition Governance (SONGO) | Maternal & child nutrition | a. Counselling b. Home visit | a. Women of reproductive age b. Adolescent girls | a. Courtyard b. Household | a. https://www.rdrsbangladesh.org/page/program/agriculture_food_security_and_climate_change b. https://reliefweb.int/report/bangladesh/songo-continues-support-beneficiaries-amid-covid-19 |
| 34 | 2019-2023 | Urban Primary Health Care Services Delivery Project –II | Maternal & child health and nutrition | Counselling | a. Pregnant women b. Adolescent girls c. Families having U-5 children | Household | https://www.ahsaniamission.org.bd/urban-primary-health-care-services-delivery-project-ii-2/ |
| 35 | 2023-2027 | Strengthening the Maternal and Neonatal Health System in Rangpur | Prenatal care, skilled birth attendance, postnatal care, family planning, and emergency obstetric care |  | a. Pregnant mothers b. Newborns c. Married women of reproductive age d. Adolescents e. Husbands and in-laws |  | a. https://bangladesh.savethechildren.net/news/save-children-and-koica-collaborate-strengthen-maternal-and-neonatal-health-rangpur-bangladesh#:~:text=20%20June%202023%2C%20Dhaka%3A%20Save,and%20newborn%20mortality%20in%20the b. https://www.dhakatribune.com/bangladesh/nation/285521/10m-project-undertaken-to-strengthen-maternal |
| 36 | 2012-2018 | Aponjon’s mHealth service | Maternal, newborn, and child health | a. Mobile incentive payments to community health agents b. SMS or voice messages c. Aponjon counseling line to talk directly to the doctors (24 hours services) d. Home visit e. Shogorbha mobile app | a. Expecting and new mothers with a child less than one year old b. Family members (husband, mother-in-law, mother and others) c. Community health agents | a. Mobile phone b. Household | a. https://pdf.usaid.gov/pdf_docs/PA00KV7Z.pdf b. https://pdf.usaid.gov/pdf_docs/PA00JV1F.pdf c. https://www.dhakatribune.com/bangladesh/bangladesh-others/145471/how-sms-changed-the-lives-of-millions-of-mothers |
| 37 | 2023- | Reaching Every Mother and Newborn (REMN) programme | Maternal and neonatal health care | a. Consultations and capacity-building activities for healthcare workers b. Counselling c. Courtyard meeting d. Publicity using loud speakers | a. Pregnant and lactating mothers b. Community people c. Health care workers | a. Health Facilities b. Courtyard | https://www.bssnews.net/special-stories/144217 |
| 38 | August 2022-December 2022 | Social behavioral change communication (SBCC) campaigns | ANC | Social media campaign (short videos and animations) | a. Women of reproductive age (18-44 years) b. Men | Facebook and Instagram | https://www.care.org/news-and-stories/ideas/care-bangladesh-drives-social-behavioral-change-in-access-to-antenatal-care/ |

**Table A.2 Description of the covariates**

| **Factor level** | **Factors** | **Categories** | **Description** |
| --- | --- | --- | --- |
| **Household characteristics** | Education of household head | None or pre-primary | No formal education or ever attended pre-school |
|  |  | Primary | At least attended class between 1 to 5 |
|  |  | Secondary | At least attended class between 6 to 10 |
|  |  | Above secondary | At least 11th class |
|  | Relationship with household head | Wife | If the respondent is household head’s wife |
|  |  | Daughter | If the respondent is household head’s daughter |
|  |  | Daughter-in-law | If the respondent is household head’s daughter-in-law |
|  |  | Other | Otherwise |
|  | Sex of household head | Male | Respondent was from a male-headed household |
|  |  | Female | Respondent was from a female-headed household |
|  | Age of household head | < 30 years | Household head’s age was thirty years or below |
|  |  | 30-39 years | Household head’s age was between 31 to 40 years |
|  |  | 40-49 years | Household head’s age was between 41 to 50 years |
|  |  | 50-59 years | Household head’s age was between 51 to 60 years |
|  |  | 60 years or above | Household head’s age was sixty-one years or above |
|  | Religion | Islam | Respondent was from a Muslim household |
|  |  | Other | Respondent was from a non-Muslim household |
|  | Wealth status | Poor | Household belonged to the first two wealth quintile groups |
|  |  | Middle | Household belonged to the third wealth quintile group |
|  |  | Rich | Household belonged to the last two wealth quintile groups |
| **Women’s characteristics** | Women's educational status | None or primary | No formal education or ever attended class between 1-5 |
|  |  | Secondary | At least attended class between 6-10 |
|  |  | Above secondary | At least 11th class |
|  | Media exposure | None | Respondent did not have exposure to any mass media |
|  |  | Less than once a week | Had exposure to any media less than once a week |
|  |  | At least once a week | Had exposure to any media at least once a week |
|  |  | Almost every day | Had exposure to any media almost every day |
|  | Age at index childbirth | <18 years | Age of the respondent at her last childbirth was below 18 years |
|  |  | 18-22 years | Age of the respondent at her last childbirth was between 18 to 22 years |
|  |  | 23-27 years | Age of the respondent at her last childbirth was between 23 to 27 years |
|  |  | 28+ years | Age of the respondent at her last childbirth was 28 years or above |
| **Women’s birth history** | Death history of children born | Had no death | Did not experience child death before the birth of the index child |
|  |  | Had at least one death | Experienced child death before the birth of the index child |
|  | Birth order of index child | First | Last child was the first birth of the respondent |
|  |  | Second | Last child was the second birth of the respondent |
|  |  | Third or higher | Last child was the third or following birth of the respondent |
|  | Sex of index child | Male | Last child was male |
|  |  | Female | Last child was female |
| **Contextual factors** | Place of residence | Rural | Respondent resided in a rural area |
|  |  | Urban | Respondent resided in an urban area |
|  | Administrative division | Dhaka | Respondent resided in Dhaka division |
|  |  | Barishal | Respondent resided in Barisal division |
|  |  | Chattogram | Respondent resided in Chattogram division |
|  |  | Khulna | Respondent resided in Khulna division |
|  |  | Mymensingh | Respondent resided in Mymensingh division |
|  |  | Rajshahi | Respondent resided in Rajshahi division |
|  |  | Rangpur | Respondent resided in Rangpur division |
|  |  | Sylhet | Respondent resided in Sylhet division |

**Table A.3 Prevalence of antenatal care (ANC) uptake and institutional birth across sociodemographic factors**

| **Factors** | **ANC uptake** | | | **IDS uptake** | |
| --- | --- | --- | --- | --- | --- |
|  | **No ANC visits** | **1-3 visits** | **At least 4 visits** | **No** | **Yes** |
| **Relationship with household head** |  |  |  |  |  |
| Wife | 19.5 | 45.6 | 34.9 | 50.6 | 49.4 |
| Daughter | 14.4 | 40.0 | 45.6 | 36.4 | 63.6 |
| Daughter in law | 12.8 | 46.8 | 40.4 | 38.6 | 61.4 |
| Other | 9.9 | 47.7 | 42.4 | 34.7 | 65.3 |
| **Sex of household head** |  |  |  |  |  |
| Male | 17.4 | 46.0 | 36.5 | 46.7 | 53.3 |
| Female | 13.1 | 40.7 | 46.1 | 40.5 | 59.5 |
| **Age of household head** |  |  |  |  |  |
| < 30 years | 17.0 | 51.5 | 31.5 | 52.2 | 47.8 |
| 30-39 years | 18.5 | 44.8 | 36.7 | 48.4 | 51.6 |
| 40-49 years | 21.0 | 42.8 | 36.1 | 49.5 | 50.5 |
| 50-59 years | 16.5 | 44.7 | 38.7 | 42.2 | 57.8 |
| 60 years or above | 12.1 | 46.3 | 41.6 | 37.6 | 62.4 |
| **Religion of household head** |  |  |  |  |  |
| Islam | 17.2 | 46.2 | 36.5 | 47.6 | 52.4 |
| Other | 17.8 | 41.3 | 41.0 | 34.5 | 65.5 |
| **Wealth status** |  |  |  |  |  |
| Poor | 29.5 | 50.6 | 19.9 | 66.5 | 33.5 |
| Middle | 15.1 | 53.5 | 31.5 | 45.5 | 54.5 |
| Rich | 5.9 | 37.4 | 56.7 | 26.7 | 73.3 |
| **Women's educational status** |  |  |  |  |  |
| No or primary | 30.6 | 47.9 | 21.5 | 67.2 | 32.8 |
| Secondary | 12.9 | 48.7 | 38.4 | 42.6 | 57.4 |
| Above secondary | 5.0 | 33.9 | 61.1 | 19.3 | 80.7 |
| **Media exposure** |  |  |  |  |  |
| None | 28.0 | 49.4 | 22.6 | 64.0 | 36.0 |
| Less than once a week | 22.7 | 51.1 | 26.2 | 52.8 | 47.2 |
| At least once a week | 18.2 | 50.1 | 31.7 | 51.8 | 48.2 |
| Almost everyday | 9.5 | 42.3 | 48.3 | 33.4 | 66.6 |
| **Age at index childbirth** |  |  |  |  |  |
| <18 years | 12.8 | 53.9 | 33.3 | 46.2 | 53.8 |
| 18-22 years | 14.6 | 49.9 | 35.5 | 43.6 | 56.4 |
| 23-27 years | 15.9 | 44.1 | 40.0 | 45.5 | 54.5 |
| 28+ years | 22.6 | 41.1 | 36.3 | 50.7 | 49.3 |
| **Number of ANC visits** |  |  |  |  |  |
| None | - | - | - | 80.5 | 19.5 |
| 1-3 | - | - | - | 51.8 | 48.2 |
| At least four | - | - | - | 24.0 | 76.0 |
| **Death history of children born** |  |  |  |  |  |
| Had no death | 16.5 | 46.0 | 37.5 | 45.9 | 54.1 |
| Had at least one death | 26.2 | 43.4 | 30.4 | 53.8 | 46.2 |
| **Birth order** |  |  |  |  |  |
| First | 10.6 | 47.1 | 42.2 | 35.5 | 64.5 |
| Secondary | 15.6 | 45.2 | 39.2 | 44.7 | 55.3 |
| Third or higher | 26.9 | 45.0 | 28.2 | 61.3 | 38.7 |
| **Sex of index child** |  |  |  |  |  |
| Male | 17.1 | 45.6 | 37.4 | 44.9 | 55.1 |
| Female | 17.5 | 46.1 | 36.4 | 48.3 | 51.7 |
| **Place of residence** |  |  |  |  |  |
| Rural | 19.7 | 48.4 | 31.9 | 50.5 | 49.5 |
| Urban | 8.9 | 36.5 | 54.6 | 32.3 | 67.7 |
| **Administrative division** |  |  |  |  |  |
| Dhaka | 12.7 | 44.6 | 42.7 | 37.6 | 62.4 |
| Barishal | 20.2 | 51.6 | 28.2 | 62.5 | 37.5 |
| Chattogram | 17.5 | 46.6 | 35.9 | 48.7 | 51.3 |
| Khulna | 7.6 | 45.0 | 47.4 | 28.5 | 71.5 |
| Mymensingh | 30.0 | 46.7 | 23.3 | 66.1 | 33.9 |
| Rajshahi | 18.8 | 47.0 | 34.1 | 42.8 | 57.2 |
| Rangpur | 16.6 | 44.4 | 38.9 | 50.4 | 49.6 |
| Sylhet | 26.5 | 43.7 | 29.8 | 59.7 | 40.3 |

Note: IDS, Institutional delivery services

**Table A.4 Association of household head’s education with at least four ANC uptake: Results from mixed-effect multivariable logistic regression**

| **Factors** | **HHs’ education** | | **HHs’ education + confounders** | | **HHs’ education + confounders + covariates** | |
| --- | --- | --- | --- | --- | --- | --- |
|  | **UOR** | **95% CI** | **AOR** | **95% CI** | **AOR** | **95% CI** |
| **Education of household**  **head** |  |  |  |  |  |  |
| Above secondary | Reference |  | Reference |  | Reference |  |
| Secondary | 0.41^***^ | [0.30,0.56] | 0.70^**^ | [0.51,0.97] | 0.74^*^ | [0.53,1.03] |
| Primary | 0.22^***^ | [0.16,0.31] | 0.55^***^ | [0.39,0.79] | 0.61^***^ | [0.42,0.87] |
| No or pre-primary | 0.19^***^ | [0.13,0.26] | 0.52^***^ | [0.36,0.77] | 0.57^***^ | [0.38,0.86] |
| **Sex of household head** |  |  |  |  |  |  |
| Male |  |  | Reference |  | Reference |  |
| Female |  |  | 1.16 | [0.70,1.93] | 1.21 | [0.70,2.08] |
| **Religion of household**  **head** |  |  |  |  |  |  |
| Islam |  |  | Reference |  | Reference |  |
| Others |  |  | 1.26 | [0.87,1.81] | 1.11 | [0.76,1.60] |
| **Wealth status** |  |  |  |  |  |  |
| Poor |  |  | Reference |  | Reference |  |
| Middle |  |  | 1.50^***^ | [1.17,1.93] | 1.33^**^ | [1.03,1.71] |
| Rich |  |  | 3.33^***^ | [2.61,4.25] | 2.89^***^ | [2.19,3.81] |
| **Women's educational**  **status** |  |  |  |  |  |  |
| No or primary |  |  | Reference |  | Reference |  |
| Secondary |  |  | 1.78^***^ | [1.38,2.28] | 1.65^***^ | [1.27,2.16] |
| Above secondary |  |  | 3.09^***^ | [2.14,4.45] | 2.58^***^ | [1.72,3.88] |
| **Place of residence** |  |  |  |  |  |  |
| Rural |  |  | Reference |  | Reference |  |
| Urban |  |  | 1.61^***^ | [1.26,2.06] | 1.60^***^ | [1.24,2.06] |
| **Relationship with**  **household head** |  |  |  |  |  |  |
| Spouse |  |  |  |  | Reference |  |
| Daughter |  |  |  |  | 1.07 | [0.56,2.04] |
| Daughter in law |  |  |  |  | 0.96 | [0.57,1.63] |
| Other |  |  |  |  | 0.82 | [0.45,1.51] |
| **Age of household head** |  |  |  |  |  |  |
| <30 |  |  |  |  | Reference |  |
| 30-39 |  |  |  |  | 1.13 | [0.80,1.58] |
| 40-49 |  |  |  |  | 1.12 | [0.74,1.71] |
| 50-59 |  |  |  |  | 1.1 | [0.64,1.89] |
| 60+ |  |  |  |  | 1.15 | [0.62,2.14] |
| **Media exposure** |  |  |  |  |  |  |
| None |  |  |  |  | Reference |  |
| <1 |  |  |  |  | 0.94 | [0.59,1.47] |
| At least 1 |  |  |  |  | 1.31 | [0.93,1.84] |
| Almost every day |  |  |  |  | 1.63^***^ | [1.29,2.06] |
| **Age at index childbirth** |  |  |  |  |  |  |
| <18 |  |  |  |  | Reference |  |
| 18-22 |  |  |  |  | 1.03 | [0.68,1.56] |
| 23-27 |  |  |  |  | 1.55^*^ | [0.98,2.45] |
| 28+ |  |  |  |  | 1.85^**^ | [1.10,3.09] |
| **Death history of children born** |  |  |  |  |  |  |
| Had no death |  |  |  |  | Reference |  |
| Had at least one death |  |  |  |  | 1.45^*^ | [0.97,2.16] |
| **Birth order of index child** |  |  |  |  |  |  |
| First |  |  |  |  | Reference |  |
| Second |  |  |  |  | 0.69^***^ | [0.52,0.91] |
| Third or higher |  |  |  |  | 0.46^***^ | [0.31,0.68] |
| **Sex of index child** |  |  |  |  |  |  |
| Male |  |  |  |  | Reference |  |
| Female |  |  |  |  | 0.97 | [0.80,1.17] |
| **Administrative division** |  |  |  |  |  |  |
| Dhaka |  |  |  |  | Reference |  |
| Barisal |  |  |  |  | 1.12 | [0.82,1.53] |
| Chattogram |  |  |  |  | 1.02 | [0.74,1.40] |
| Khulna |  |  |  |  | 1.90^***^ | [1.44,2.51] |
| Mymensingh |  |  |  |  | 0.82 | [0.51,1.30] |
| Rajshahi |  |  |  |  | 1.09 | [0.77,1.53] |
| Rangpur |  |  |  |  | 1.84^***^ | [1.36,2.49] |
| Sylhet |  |  |  |  | 1.08 | [0.69,1.68] |
| **Intra cluster correlation** | 24.3% |  | 18.7% |  | 18.0% |  |
| **Observations** | **8943** |  | **8943** |  | **8943** |  |

Note: * p-value < 0.10, ** p-value < 0.05, *** p-value < 0.01, UOR: Unadjusted odds ratio, AOR: Adjusted odds ratio, CI: Confidence interval

**Table A.5 Association of household head’s education with at facility birth: Results from mixed-effect multivariable logistic regression**

| **Factors** | **HHs’ education** | | **HHs’ education + confounders** | | **HHs’ education + confounders + covariates** | |
| --- | --- | --- | --- | --- | --- | --- |
|  | **UOR** | **95% CI** | **AOR** | **95% CI** | **AOR** | **95% CI** |
| **Education of household head** |  |  |  |  |  |  |
| Above secondary | Reference |  | Reference |  | Reference |  |
| Secondary | 0.38^***^ | [0.26,0.56] | 0.70^*^ | [0.47,1.05] | 0.72 | [0.47,1.12] |
| Primary | 0.19^***^ | [0.13,0.27] | 0.53^***^ | [0.36,0.80] | 0.56^**^ | [0.35,0.87] |
| No or pre-primary | 0.14^***^ | [0.10,0.20] | 0.45^***^ | [0.29,0.68] | 0.44^***^ | [0.27,0.71] |
| **Sex of household head** |  |  |  |  |  |  |
| Male |  |  | Reference |  | Reference |  |
| Female |  |  | 1.02 | [0.61,1.72] | 0.88 | [0.52,1.48] |
| **Religion of household head** |  |  |  |  |  |  |
| Islam |  |  | Reference |  | Reference |  |
| Others |  |  | 2.20^***^ | [1.57,3.08] | 2.01^***^ | [1.42,2.84] |
| **Wealth status** |  |  |  |  |  |  |
| Poor |  |  | Reference |  | Reference |  |
| Middle |  |  | 1.82^***^ | [1.46,2.28] | 1.35^**^ | [1.05,1.72] |
| Rich |  |  | 3.46^***^ | [2.72,4.41] | 1.99^***^ | [1.48,2.67] |
| **Women's educational status** |  |  |  |  |  |  |
| No or primary |  |  | Reference |  | Reference |  |
| Secondary |  |  | 2.16^***^ | [1.70,2.75] | 1.55^***^ | [1.18,2.04] |
| Above secondary |  |  | 4.53^***^ | [3.14,6.54] | 2.43^***^ | [1.59,3.74] |
| **Place of residence** |  |  |  |  |  |  |
| Rural |  |  | Reference |  | Reference |  |
| Urban |  |  | 1.32^**^ | [1.00,1.74] | 1.16 | [0.87,1.54] |
| **Relationship with household head** |  |  |  |  |  |  |
| Spouse |  |  |  |  | Reference |  |
| Daughter |  |  |  |  | 1.23 | [0.56,2.68] |
| Daughter in law |  |  |  |  | 1.15 | [0.59,2.23] |
| Other |  |  |  |  | 1.18 | [0.62,2.27] |
| **Age of household head** |  |  |  |  |  |  |
| <30 |  |  |  |  | Reference |  |
| 30-39 |  |  |  |  | 1.24 | [0.91,1.68] |
| 40-49 |  |  |  |  | 1.22 | [0.81,1.84] |
| 50-59 |  |  |  |  | 1.26 | [0.68,2.34] |
| 60+ |  |  |  |  | 1.31 | [0.65,2.64] |
| **Media exposure** |  |  |  |  |  |  |
| None |  |  |  |  | Reference |  |
| <1 |  |  |  |  | 1.26 | [0.84,1.88] |
| At least 1 |  |  |  |  | 1.18 | [0.85,1.62] |
| Almost every day |  |  |  |  | 1.48^***^ | [1.16,1.88] |
| **Age at index childbirth** |  |  |  |  |  |  |
| <18 |  |  |  |  | Reference |  |
| 18-22 |  |  |  |  | 1.14 | [0.74,1.76] |
| 23-27 |  |  |  |  | 1.45 | [0.88,2.40] |
| 28+ |  |  |  |  | 1.77^**^ | [1.04,3.01] |
| **Number of ANC visits** |  |  |  |  |  |  |
| None |  |  |  |  | Reference |  |
| 1-3 |  |  |  |  | 2.74^***^ | [2.07,3.64] |
| At least four |  |  |  |  | 6.73^***^ | [4.86,9.32] |
| **Death history of children born** |  |  |  |  |  |  |
| Had no death |  |  |  |  | Reference |  |
| Had at least one death |  |  |  |  | 1.85^***^ | [1.31,2.60] |
| **Birth order of index child** |  |  |  |  |  |  |
| First |  |  |  |  | Reference |  |
| Second |  |  |  |  | 0.54^***^ | [0.41,0.73] |
| Third or higher |  |  |  |  | 0.31^***^ | [0.21,0.46] |
| **Sex of index child** |  |  |  |  |  |  |
| Male |  |  |  |  | Reference |  |
| Female |  |  |  |  | 0.83^*^ | [0.68,1.02] |
| **Administrative division** |  |  |  |  |  |  |
| Dhaka |  |  |  |  | Reference |  |
| Barisal |  |  |  |  | 0.52^***^ | [0.38,0.71] |
| Chattogram |  |  |  |  | 0.72^**^ | [0.52,0.99] |
| Khulna |  |  |  |  | 1.77^***^ | [1.28,2.45] |
| Mymensingh |  |  |  |  | 0.52^***^ | [0.35,0.78] |
| Rajshahi |  |  |  |  | 1.16 | [0.81,1.65] |
| Rangpur |  |  |  |  | 0.75^*^ | [0.53,1.06] |
| Sylhet |  |  |  |  | 0.63^**^ | [0.40,0.97] |
| **Intra cluster correlation** | 29.0% |  | 21.3% |  | 21.3% |  |
| **Observations** | **8943** |  | **8943** |  | **8943** |  |

Note: * p-value < 0.10, ** p-value < 0.05, *** p-value < 0.01, UOR: Unadjusted odds ratio, AOR: Adjusted odds ratio, CI: Confidence interval

**Table A.6 Interaction of household head's education with number of ANC visits**

| **Factors** | **AOR** | **95% CI** |
| --- | --- | --- |
| **Education of household head** |  |  |
| Above secondary | Reference |  |
| Secondary | 0.59 | [0.27,1.30] |
| Primary | 0.38^**^ | [0.18,0.81] |
| No or pre-primary | 0.34^***^ | [0.16,0.73] |
| **ANC visits** |  |  |
| None | Reference |  |
| 1-3 | 1.75 | [0.81,3.78] |
| 4+ | 6.02^***^ | [2.83,12.79] |
| **Interaction (household head's education* ANC visits)** |  |  |
| Secondary education, 1-3 ANC visits | 1.41 | [0.59,3.36] |
| Primary education, 1-3 ANC visits | 1.82 | [0.79,4.20] |
| No or pre-primary education, 1-3 ANC visits | 1.59 | [0.69,3.66] |
| Secondary education, 4+ ANC visits | 1.11 | [0.47,2.62] |
| Primary education, 4+ ANC visits | 1.20 | [0.52,2.75] |
| No or pre-primary education, 4+ ANC visits | 1.00 | [0.44,2.28] |
| **Relationship with household head** |  |  |
| Wife | Reference |  |
| Daughter | 1.23 | [0.77,1.96] |
| Daughter in law | 1.15 | [0.80,1.65] |
| Other | 1.18 | [0.80,1.75] |
| **Sex of household head** |  |  |
| Male | Reference |  |
| Female | 0.88 | [0.62,1.24] |
| **Age of household head** |  |  |
| < 30 years | Reference |  |
| 30-39 years | 1.23^**^ | [1.01,1.52] |
| 40-49 years | 1.22 | [0.93,1.60] |
| 50-59 years | 1.26 | [0.87,1.84] |
| 60 years or above | 1.31 | [0.86,1.98] |
| **Religion of household head** |  |  |
| Islam | Reference |  |
| Other | 2.02^***^ | [1.60,2.54] |
| **Wealth status** |  |  |
| Poor | Reference |  |
| Middle | 1.35^***^ | [1.14,1.61] |
| Rich | 1.99^***^ | [1.65,2.41] |
| **Women's educational status** |  |  |
| No or primary | Reference |  |
| Secondary | 1.55^***^ | [1.32,1.81] |
| Above secondary | 2.41^***^ | [1.87,3.09] |
| **Media exposure** |  |  |
| None | Reference |  |
| Less than once a week | 1.26 | [0.94,1.68] |
| At least once a week | 1.18 | [0.94,1.48] |
| Almost everyday | 1.47^***^ | [1.26,1.72] |
| **Age at index childbirth** |  |  |
| <18 years | Reference |  |
| 18-22 years | 1.14 | [0.88,1.49] |
| 23-27 years | 1.44^**^ | [1.07,1.95] |
| 28+ years | 1.75^***^ | [1.26,2.44] |
| **Death history of children born** |  |  |
| Had no death | Reference |  |
| Had at least one death | 1.86^***^ | [1.48,2.33] |
| **Birth order of index child** |  |  |
| First | Reference |  |
| Second | 0.55^***^ | [0.45,0.66] |
| Third or higher | 0.31^***^ | [0.24,0.40] |
| **Sex of index child** |  |  |
| Male | Reference |  |
| Female | 0.83^***^ | [0.73,0.94] |
| **Place of residence** |  |  |
| Rural | Reference |  |
| Urban | 1.15 | [0.96,1.38] |
| **Administrative division** |  |  |
| Dhaka | Reference |  |
| Barishal | 0.52^***^ | [0.40,0.67] |
| Chattogram | 0.72^***^ | [0.58,0.88] |
| Khulna | 1.78^***^ | [1.40,2.26] |
| Mymensingh | 0.52^***^ | [0.39,0.68] |
| Rajshahi | 1.16 | [0.92,1.47] |
| Rangpur | 0.75^**^ | [0.59,0.97] |
| Sylhet | 0.62^***^ | [0.47,0.82] |
| **Intra cluster correlation** | 18.6 |  |

Note: * p-value < 0.10, ** p-value < 0.05, *** p-value < 0.01, UOR: Unadjusted odds ratio, AOR: Adjusted odds ratio, CI: Confidence interval

**Table A.7 Interventions implemented during 2000 to 2023 to improve school enrollment**

| **Serial** | **Project period** | **Title of the program/intervention** | **Type of intervention** | **Level of education** | **Intervention recipient** | **Implementer** | **Source** |
| --- | --- | --- | --- | --- | --- | --- | --- |
| 1 | 1993-2001 | Food for Education (FFE) Program | Free monthly ration (rice/wheat) | Primary | Both | Govt. | a. https://www.researchgate.net/publication/5056361_The_Food_For_Education_program_in_Bangladesh#:~:text=The%20authors%20first%20examine%20the,%2C%20however%2C%20remains%20a%20problem. b. https://www.researchgate.net/publication/44836609_The_Bangladesh_Primary_Education_Stipend_Project_a_descriptive_analysis |
| 2 | 1994-2001 | Female Secondary School Assistance Project (FSSAP) (Phase I) | Stipend and tuition subsidy | Secondary | Girls | Govt. | a. https://unesdoc.unesco.org/ark:/48223/pf0000146803 b. https://www.adb.org/publications/female-secondary-stipend-assistance-program-bangladesh c. https://papers.ssrn.com/sol3/papers.cfm?abstract_id=3372022#:~:text=Literacy%20rate%20of%20women,-Table%201%3A%20Female&text=But%20in%202010%2C%20it%20was,female%20literacy%20rate%20by%2012.1%25. |
| 3 | 2002-2007 | Female Secondary School Assistance Project (FSSAP) (Phase II) | Stipend and tuition subsidy | Secondary | Girls | Govt. | a. https://unesdoc.unesco.org/ark:/48223/pf0000146803 b. https://papers.ssrn.com/sol3/papers.cfm?abstract_id=3372022#:~:text=Literacy%20rate%20of%20women,-Table%201%3A%20Female&text=But%20in%202010%2C%20it%20was,female%20literacy%20rate%20by%2012.1%25. |
| 4 | 1994-2005 | Female Secondary Stipend Project (FSSP) (Phase I) | Stipend and tuition subsidy | Secondary | Girls | Govt. | a. https://imed.portal.gov.bd/sites/default/files/files/imed.portal.gov.bd/page/e773d5bf_182e_4fc5_a856_dfd3c8d05ced/Female.pdf b. https://www.adb.org/publications/female-secondary-stipend-assistance-program-bangladesh |
| 5 | 2005-2008 | Female Secondary Stipend Project (FSSP) (Phase II) | Stipend and tuition subsidy | Secondary | Girls | Govt. | <https://imed.portal.gov.bd/sites/default/files/files/imed.portal.gov.bd/page/e773d5bf_182e_4fc5_a856_dfd3c8d05ced/Female.pdf> |
| 6 | 1993-2002 | Secondary Education Development Project (SEDP) | Stipend and tuition subsidy | Secondary | Girls | Govt. | a. https://unesdoc.unesco.org/ark:/48223/pf0000146803 b. https://www.adb.org/publications/female-secondary-stipend-assistance-program-bangladesh |
| 7 | 1999-2006 | Secondary Education Sector Improvement Project (SESIP) | Stipend and tuition subsidy | Secondary | Girls | Govt. | a. https://unesdoc.unesco.org/ark:/48223/pf0000146803 b. https://www.adb.org/publications/female-secondary-stipend-assistance-program-bangladesh |
| 8 | 1997-2002 | Female Secondary Education Stipend Project (FSEP) | Stipend and tuition subsidy | Secondary | Girls | Govt. | a. https://unesdoc.unesco.org/ark:/48223/pf0000146803 b. https://www.adb.org/publications/female-secondary-stipend-assistance-program-bangladesh |
| 9 | 2002-2008 | Primary Education Stipend Project (PESP) (Phase I) | Stipend | Primary | Both | Govt. | https://www.researchgate.net/publication/44836609_The_Bangladesh_Primary_Education_Stipend_Project_a_descriptive_analysis |
| 10 | 2009-2015 | Primary Education Stipend Project (PESP) (Phase II) | Stipend | Primary | Both | Govt. | a. https://planipolis.iiep.unesco.org/sites/default/files/ressources/bangladesh_pedpii_features.pdf b. https://dpe.portal.gov.bd/sites/default/files/files/dpe.portal.gov.bd/page/093c72ab_a76a_4b67_bb19_df382677bebe/PEDP-3%20Brief%20(Revised).pdf |
| 11 | 2016-2021 | Primary Education Stipend Project (PESP) (Phase III) | Stipend | Primary | Both | Govt. | <https://dpe.portal.gov.bd/sites/default/files/files/dpe.portal.gov.bd/page/093c72ab_a76a_4b67_bb19_df382677bebe/PEDP-3%20Brief%20(Revised).pdf> |
| 12 | 2005-2012 | Reaching Out-of-School Children (ROSC) Project (Phase I) | Establishing Ananda Schools (improved access, quality and efficiency) | Primary | Both | Both | <https://papers.ssrn.com/sol3/papers.cfm?abstract_id=3372022#:~:text=Literacy%20rate%20of%20women,-Table%201%3A%20Female&text=But%20in%202010%2C%20it%20was,female%20literacy%20rate%20by%2012.1%25.> |
| 13 | 2013-2017 | Reaching Out-of-School Children (ROSC) Project (Phase II) | Establishing Ananda Schools (improved access, quality and efficiency) | Primary | Both | NGO | https://papers.ssrn.com/sol3/papers.cfm?abstract_id=3372022#:~:text=Literacy%20rate%20of%20women,-Table%201%3A%20Female&text=But%20in%202010%2C%20it%20was,female%20literacy%20rate%20by%2012.1%25. |
| 14 | 2008-2017 | Secondary Education Quality and Access Enhancement Project (SEQAEP) | Stipend and other allowance (tuition fee, Exam fee) | Secondary | Both | Govt. | a. https://www.worldbank.org/en/results/2014/03/11/bangladesh-incentivizing-secondary-education b. https://www.adb.org/publications/female-secondary-stipend-assistance-program-bangladesh c.https://papers.ssrn.com/sol3/papers.cfm?abstract_id=3372022#:~:text=Literacy%20rate%20of%20women,Table%201%3A%20Female&text=But%20in%202010%2C%20it%20was,female%20literacy%20rate%20by%2012.1%25. |
| 15 | 2005-2008 | Higher Secondary Female Stipend Project (HSSP) (Phase III) | Stipend and allowances | Higher Secondary | Girls | Govt. | <https://imed.portal.gov.bd/sites/default/files/files/imed.portal.gov.bd/page/e773d5bf_182e_4fc5_a856_dfd3c8d05ced/Higher%20%281%29.pdf> |
| 16 | 2009-2014 | Higher Secondary Female Stipend Project (HSSP) (Phase IV) | Stipend and allowances | Higher Secondary | Girls | Govt. | https://papers.ssrn.com/sol3/papers.cfm?abstract_id=3372022#:~:text=Literacy%20rate%20of%20women,-Table%201%3A%20Female&text=But%20in%202010%2C%20it%20was,female%20literacy%20rate%20by%2012.1%25. |
| 17 | 2009-2012 | Secondary Education Stipend Project (SESP) (Phase I) | Stipend and other allowance (tuition fee, Exam fee) | Secondary | Both (10% male and 30% female) | Govt. | a.https://www.adb.org/publications/female-secondary-stipend-assistance-program-bangladesh b.https://papers.ssrn.com/sol3/papers.cfm?abstract_id=3372022#:~:text=Literacy%20rate%20of%20women,-Table%201%3A%20Female&text=But%20in%202010%2C%20it%20was,female%20literacy%20rate%20by%2012.1%25. |
| 18 | 2014-2017 | Secondary Education Stipend Project (SESP) (Phase II) | Stipend and other allowance (tuition fee, Exam fee) | Secondary | Both (10% male and 30% female) | Govt. | https://www.adb.org/publications/female-secondary-stipend-assistance-program-bangladesh |
| 19 | 2010-2015 | School Feeding Program | Fortified biscuits | Primary | Both | Govt. | <https://www.thedailystar.net/news/bangladesh/news/mid-day-meals-likely-govt-primary-school-students-july-3247876> |
| 20 | 2023-2026 | Government School Feeding Program (Phase-1) | A diversified food basket comprising fortified biscuits, buns, fruits (banana), eggs and ultra-high temperature milk and in some selected upazilas will receive a nutritious cooked meal (fortified rice, red lentils, soybean oil, vegetables and eggs) | Primary | Both | Govt. | <https://www.tbsnews.net/bangladesh/education/school-feeding-programme-targets-35-lakh-kids-poverty-prone-areas-675518> |
| 21 | 2015-2022 | School Feeding Program | Fortified biscuits and in some selected upazilas received a nutritious cooked meal (fortified rice, red lentils, soybean oil, eggs and fresh vegetables) | Primary | Both | Govt. | https://www.tbsnews.net/bangladesh/education/school-feeding-programme-targets-35-lakh-kids-poverty-prone-areas-675518 |
| 22 | 2001-2009 | School Feeding Program | High-energy biscuits | Primary | Both | NGO | <https://documents.wfp.org/stellent/groups/public/documents/tor/wfp235770.pdf> |
| 23 | 2017- | Mayer Hasi | Stipend using mobile Banking | Primary | Both | Govt. | https://www.dhakatribune.com/bangladesh/education/15547/pm-launches-stipend-distribution-through-mobile |
| 24 | 2013-2023 | Secondary Education Sector Investment Program (SESIP) | Developing teaching program using ICT, Stipend and other allowance (tuition fee, Exam fee) | Secondary | Both (20% male and 30% female) | Govt. | a. https://www.adb.org/sites/default/files/project-documents/44213/44213-016-44213-017-emr-en_3.pdf b. https://www.adb.org/publications/female-secondary-stipend-assistance-program-bangladesh |
| 25 | 2014- | Higher Secondary Stipend Project (HSSP) | Stipend and other allowance (tuition fee, Exam fee, books) | Higher Secondary | Both (10% male and 40% female) | Govt. | https://www.adb.org/publications/female-secondary-stipend-assistance-program-bangladesh |
| 26 | 2007-2013 | Secondary Education Sector Development Program (SESDP) | Stipend | Secondary | Both (10% male and 30% female) | Govt. | a. https://www.scribd.com/document/170466763/Bangladesh-Secondary-Education-Sector-Development-Program b. https://www.adb.org/publications/female-secondary-stipend-assistance-program-bangladesh |
| 27 | 2018-2022 | Transforming Secondary Education for Results (TSER) Program | Stipends and school grants | Secondary and higher secondary | Girls | Govt. | a. https://www.worldbank.org/en/news/press-release/2017/12/18/world-bank-helps-bangladesh-improve-secondary-education-benefiting-13-million-students b. https://borgenproject.org/girls-education-in-bangladesh/ c. https://documents1.worldbank.org/curated/en/099230503272328115/pdf/P17848701d4af10008f620555f70a06c7d.pdf |
| 28 | 2010- | Free Text Book Distribution Program (FTBDP) | Free textbooks | Pre-primary, primary, secondary, Ebtedayee and SSC level students | Both | Govt. | <https://www.dhakatribune.com/bangladesh/301681/pm-hasina-inaugurates-nationwide-free-textbook> |
| 29 | 1985- | BRAC Education Programme | Providing pre-primary school education, non-formal primary education | Pre-primary and primary | Both (special focus on girls in primary level) | NGO | <https://uil.unesco.org/case-study/effective-practices-database-litbase-0/brac-education-programme-bangladesh> |
| 30 | 2022- | Out-of-School Children Education Program | Providing non formal primary education, stipend, free books, notebooks, pens, dresses and bags | Primary | Both (age: 8-14 years) | NGO | https://www.tbsnews.net/bangladesh/education/education-programme-10-lakh-out-school-children-faces-hiccups-510610 |
| 31 | 2018-2022 | Empowering Girls Through Education | Assisting the transition of girls from primary to secondary education through introducing interactive, and child-friendly e-contents and awareness building | Class-4, 5, & 6 | Girls | NGO | a. https://friendship.ngo/ege-closing-ceremony/,  b. https://www.campebd.org/Details.php?DetId=442 |
| 32 | 2021-2022 | STEM Education Program | Nationwide STEM (science, technology, engineering, and math) competitions, career counselling | University level (age: 18-25) | Girls | NGO | https://thefinancialexpress.com.bd/education/initiative-launched-to-encourage-bangladeshi-women-girls-pursue-career-in-science-1615292119 |
| 33 | 1994- | Child Education Program | Establishing learning centers and introducing an innovative, ‘active learning method’ | Primary | Both | NGO | https://fivdb.org/history |
| 34 | 2023-2027 | Shobai Miley Shikhi | Improve learning opportunities (hard-to-reach areas) | Primary | Both (focus on those with disabilities or special needs) | NGO | https://www.dhakatribune.com/bangladesh/education/298634/bangladesh-us-launch-18-million-inclusive |
| 35 | 1992-1994 | Integrated Non-Formal Education Programme (INFEP) | Providing non-formal education | Non-formal education | Both (children, adolescents and adults) | Govt. | https://unesdoc.unesco.org/ark:/48223/pf0000113253 |
| 36 | 1997-2003 | Primary education development programme (PEDP I) | Improving quality and access in primary education | Primary | Both | Govt. | https://dpe.portal.gov.bd/sites/default/files/files/dpe.portal.gov.bd/page/093c72ab_a76a_4b67_bb19_df382677bebe/PEDP-3%20Brief%20(Revised).pdf |
| 37 | 2004-2011 | Primary education development programme (PEDP II) | Improving quality and access in primary education | Primary | Both | Govt. | a. https://dpe.portal.gov.bd/sites/default/files/files/dpe.portal.gov.bd/page/093c72ab_a76a_4b67_bb19_df382677bebe/PEDP-3%20Brief%20(Revised).pdf b. https://planipolis.iiep.unesco.org/2004/second-primary-education-development-program-pedp-ii-4358 |
| 38 | 2011-2017 | Primary education development programme (PEDP III) | Establishing an efficient, inclusive and equitable primary education system (stipend, text book development, second chance education etc.), free pre-primary education | Pre-primary, primary | Both | Govt. | https://dpe.portal.gov.bd/sites/default/files/files/dpe.portal.gov.bd/page/093c72ab_a76a_4b67_bb19_df382677bebe/PEDP-3%20Brief%20(Revised).pdf |
| 39 | 2018-2023 | Primary education development programme (PEDP IV) | Universal access, equity of teaching and student learning, institutional strengthening, program planning and management | Pre-primary, primary | Both | Govt. | https://dpe.portal.gov.bd/sites/default/files/files/dpe.portal.gov.bd/publications/4321a093_51d5_4b1d_a3c3_842865746493/The%20Social%20Management%20Framework%20(SMF)%20(1).pdf |
| 40 | 2021-2025 | Learning Roots | Improved learning and development for children | Pre-primary | Both (age: 3-6 years) | NGO | https://www.wvi.org/education-programme |
| 41 | 2021-2025 | Unlock Literacy | Basic literacy and numeracy skills for children | Primary | Both (age: 6-9 years) | NGO | https://www.wvi.org/education-programme |
| 42 | 2012-2020 | English and Digital for Girls’ Education (EDGE) (Phase I) | Building English, ICT and social skills |  | Girls (age:14-19 years) | NGO | https://www.britishcouncil.org.bd/en/programmes/education/empower-bangladesh |
| 43 | 2021- | English and Digital for Girls’ Education (EDGE) (Phase II) | Building English, ICT and social skills |  | Girls (age:14-19 years) | NGO | https://www.britishcouncil.org.bd/en/programmes/education/empower-bangladesh |
| 44 | 2019-2021 | Let Us Learn Project in Sunamgon (For out of school children) | Ability Based Accelerated Learning (ABAL) approach along with Pre-Primary Education (PPE) | Pre-primary | Both (For PPE, age: 5-6 years; For ABAL, age: 8-14years) | NGO | https://www.ahsaniamission.org.bd/let-us-learn/ |
